# Early-Life Immune System Maturation in Chickens Using a Synthetic Community of Cultured Gut Bacteria

**DOI:** 10.1128/mSystems.01300-20

**Published:** 2021-05-18

**Authors:** Christian Zenner, Thomas C. A. Hitch, Thomas Riedel, Esther Wortmann, Stefan Tiede, Eva M. Buhl, Birte Abt, Klaus Neuhaus, Philippe Velge, Jörg Overmann, Bernd Kaspers, Thomas Clavel

**Affiliations:** a Department for Veterinary Sciences, Veterinary Immunology Study Group, Ludwig-Maximilians-University Munich, Munich, Germany; b Functional Microbiome Research Group, RWTH University Hospital, Aachen, Germany; c Leibniz Institute DSMZ-German Collection of Microorganisms and Cell Cultures, Braunschweig, Germany; d German Center for Infection Research (DZIF), Partner site Hannover-Braunschweig, Braunschweig, Germany; e Electron Microscopy Facility, Institute of Pathology, RWTH University Hospital Aachen, Aachen, Germany; f Core Facility Microbiome, ZIEL Institute for Food & Health, Technical University of Munich, Freising, Germany; g ISP, INRAE, Université François Rabelais de Tours, UMR 1282, Nouzilly, France; h Faculty of Life Sciences, Braunschweig University of Technology, Braunschweig, Germany; Colorado State University

**Keywords:** synthetic bacterial community, anaerobes, chicken, gut microbiome, mucosal immunology

## Abstract

The gut microbiome is crucial for both maturation of the immune system and colonization resistance against enteric pathogens. Although chicken are important domesticated animals, the impact of their gut microbiome on the immune system is understudied. Therefore, we investigated the effect of microbiome-based interventions on host mucosal immune responses. Increased levels of IgA and IgY were observed in chickens exposed to maternal feces after hatching compared with strict hygienic conditions. This was accompanied by increased gut bacterial diversity as assessed by 16S rRNA gene amplicon sequencing. Cultivation work allowed the establishment of a collection of 43 bacterial species spanning 4 phyla and 19 families, including the first cultured members of 3 novel genera and 4 novel species that were taxonomically described. This resource is available at www.dsmz.de/chibac. A synthetic community consisting of nine phylogenetically diverse and dominant species from this collection was designed and found to be moderately efficient in boosting immunoglobulin levels when provided to chickens early in life.

**IMPORTANCE** The immune system plays a crucial role in sustaining animal health. Its development is markedly influenced by early microbial colonization of the gastrointestinal tract. As chicken are fully dependent on environmental microbes after hatching, extensive hygienic measures in production facilities are detrimental to the microbiota, resulting in low colonization resistance against pathogens. To combat enteric infections, antibiotics are frequently used, which aggravates the issue by altering gut microbiota colonization. Intervention strategies based on cultured gut bacteria are proposed to influence immune responses in chicken.

## INTRODUCTION

Poultry meat is the main source of alimentary proteins for human consumption worldwide (1). Broilers and layers have been bred for high productivity traits such as growth rates, feed conversion, and egg production (2). However, infections and poor health status in contemporary, large-scale production facilities are major problems both ethically and economically. As the European Union banned the use of antibiotics as growth promoters (3) and the spread of antibiotic resistances is of real concern (4), alternatives to prevent infections and maintain healthy chicken flocks are urgently needed. High hygienic measures, including detailed disinfection plans and restrictive facility access, are meant to avoid pathogen entry into production facilities (5). However, as chicken are fully dependent on microbes taken up after hatching for colonization of their gastrointestinal tract, in contrast to mammals, which acquire parental and environmental microbes during birth, a sterile hatching environment without close contact to adult animals can strongly affect gut microbiome diversity (6).

The chicken intestinal microbiome is a diverse and complex ecosystem, including hundreds of bacterial species (7) that can metabolize food components (8, 9), support the development of immune functions (10), and protect against pathogens (11–13). The gastrointestinal tract of chicken consists of upper compartments (e.g., crop), the small intestine, ceca, and colon. The ceca are two blind pouches located between the ileum and colon. They are major sites of microbial fermentation and harbor higher microbial diversity compared to other gut regions (14), explaining why earlier cultivation studies focused on the cecum microbiota (15–17). The development of high-throughput molecular techniques has since revolutionized microbiome research. Now, combining cultivation and sequencing offers unique opportunities to study gut microbes (18, 19). In comparison to numerous studies that have investigated the gut microbiota of mice and humans (20, 21), work on domestic animals other than cows and pigs (22–24) is scarce.

With respect to intervention strategies, a study in the 1970s treated hatched chickens orally with crop and intestinal content of adult birds, successfully inhibiting a subsequent infection by Salmonella enterica serovar Infantis (25). In 1988, Goren et al. inoculated chickens with intestinal homogenates in a large longitudinal study with more than 8 million broilers that were evaluated flock-wise, demonstrating a significantly lower Salmonella incidence in treated flocks (26). In 2016, Varmuzova et al. colonized newly hatched chickens orally with cecal extracts originating from birds of different ages to promote resistance against Salmonella enteritidis. While extracts from ≥3 week-old chickens were protective, extracts from younger chickens were not (13). Moreover, Kubasova et al. reported that the contact of hatched chickens to adult hens over 24 h is mandatory for the transfer of *Bacteroidetes* and *Actinobacteria* (27). Even though these are promising results, providing complex, undefined stool material at large-scale to sustain or improve chicken health is not feasible.

Another concept of intervention is to use minimal bacterial consortia, also referred to as synthetic communities. Used in mice by Schaedler et al. in the 1960s (28), the approach consists of providing mixtures of a limited number of phylogenetically diverse and dominant cultured members of native communities, which was recently shown to confer colonization resistance against Salmonella enterica serovar Typhimurium and Clostridioides difficile in mice and human (29–31). The design and use of minimal bacterial consortia requires the existence of comprehensive collections of isolates, which have been missing in chicken. In 2020, Rychlik reviewed the composition and functions of chicken gut microbiota and stated that one of the main future challenges is “to generate an extensive collection of pure cultures of chicken gut anaerobes” (32). His group already provided 133 genomes of anaerobic bacteria isolated from the chicken gut as a solid foundation for future work (19). However, more effort is necessary to obtain a comprehensive view of chicken gut bacteria, especially anaerobic species. Recently, Crhanova et al. suggested that half of the chicken cecal microbiota members could be cultured *in vitro*, although pure isolates remain to be obtained (33).

In the present study, we combined molecular and culture-based investigations to evaluate the impact of the chicken gut microbiome on immune functions. Taxonomic and ecological insights into cultured chicken gut bacteria are provided and effects on the host were tested in colonization trials with complex and simplified microbial communities.

## RESULTS

### Early-life interventions with maternal microbiota trigger adaptive immune responses in chicken.

To test the hypothesis that the development of immune responses in chicken is driven by the gut microbiome, an intervention trial, including animals with various degrees of microbial exposure, was performed. Chickens exposed to maternal microbiota (MM) immediately after hatching were compared to specific pathogen-free (SPF) animals.

**FIG 1:**
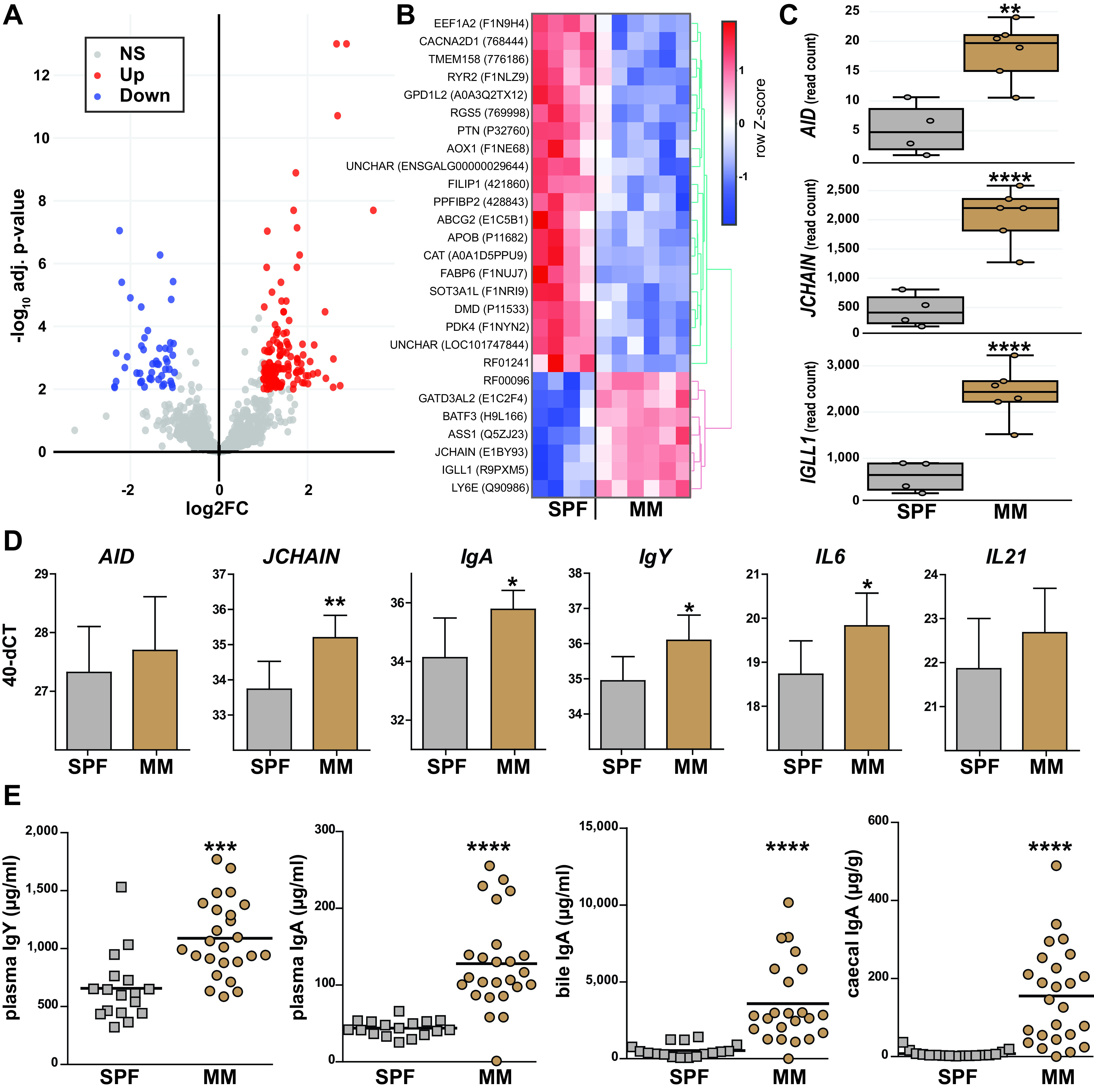
Host immune responses after fecal exposure. All readouts were generated at the age of 58 days. Chickens treated with maternal microbiota (MM) after hatch were compared to a control group kept under specific pathogen-free conditions (SPF). (A) Volcano plot of significantly and differentially regulated genes in cecal tonsil as assessed by RNA-seq (*n*_SPF_ = 4, *n*_MM_ = 6). (B) Heat map of the most significantly regulated genes (−log_10_ adjusted *P* value of >4). Uniprot accession numbers are indicated in brackets. (C) Read counts of the differentially expressed, immunologically relevant genes “activation induced cytidine deaminase” (AID), “joining chain” (JCHAIN), and “immunoglobulin variable region” (IGLL1). (D) qPCR analysis of immunologically relevant genes AID, JCHAIN, IgA, IgY, IL-6, and IL-21 (*n *= 6 in both groups). (E) Immunoglobulin concentrations as determined by quantitative ELISA (*n*_SPF_ = 17 and *n*_MM_ = 25 for IgY and IgA in plasma, and IgA in cecal content; *n*_MM_ = 23 for IgA in bile). Adjusted *P* values for RNA-seq were obtained using the Wald test, including adjustment for multiple testing (Benjamini-Hochberg); *P* values for qPCR and ELISA were obtained by Mann-Whitney U test: *, *P* < 0.05; **, *P* < 0.01; ***, *P* < 0.001; ****, *P* < 0.0001.

Gene expression in cecal tonsils, which are important mucosal immune sites in chicken, was investigated in a nontarget manner by RNA transcriptome sequencing (RNA-seq). In total, 177 genes were differentially regulated (adjusted *P* value <0.01, fold change >2) between the two colonization groups (Fig. 1A). Among the highest significantly regulated genes (−log_10_ adjusted *P* value >4), shown as a heat map in Fig. 1B, gene ontology (GO) terms related to responses to external stimuli and stress (GO:0051707, GO:0043207, GO:0009607, GO:0009605, and GO:0006950) were specific for MM chickens, while GO terms related to lipid and fatty acid metabolism, as well as oxido-reduction (GO:0006631, GO:0032787, GO:0044255, GO:0006629, and GO:0055114) were enriched in SPF controls (*P* < 0.001). The latter group was characterized by upregulated genes involved in lipid metabolism (APOB and FABP6), whereas the following, immunologically relevant genes were significantly upregulated in MM chickens: (i) JCHAIN, the joining chain of multimeric immunoglobulin (Ig) A (IgA) and M (IgM); (ii) IGLL1, which encodes the Ig variable region; and (iii) AID, activation-induced cytidine deaminase, which plays a crucial role in class switch recombination and affinity maturation of antibodies (Fig. 1C).

These results from the transcriptome analysis were confirmed using quantitative PCR (qPCR). The gene expression of AID, JCHAIN, IgA, IgY, interleukin 6 (IL-6), and interleukin 21 (IL-21) was quantified in cecal tonsils, with JCHAIN, IgA, IgY, and IL-6 significantly increased, while no changes were observed for AID and IL-21 (Fig. 1D). Importantly, quantitative Ig measurements by enzyme-linked immunosorbent assay (ELISA) in a higher number of animals (*n*_SPF_ = 17; *n*_MM_ = 25) corroborated these data obtained at the transcriptional level. Significantly larger amounts of IgA (*P* < 0.0001) and IgY (*P* = 0.0002) in plasma, and also IgA in bile and cecal content (*P* < 0.0001), were observed in MM chickens (Fig. 1E).

In summary, early fecal exposure of chickens impacted transcriptional responses within a main gut-associated lymphoid tissue and triggered higher production of antibodies.

### Fecal exposure markedly affected the gut microbiota.

**FIG 2:**
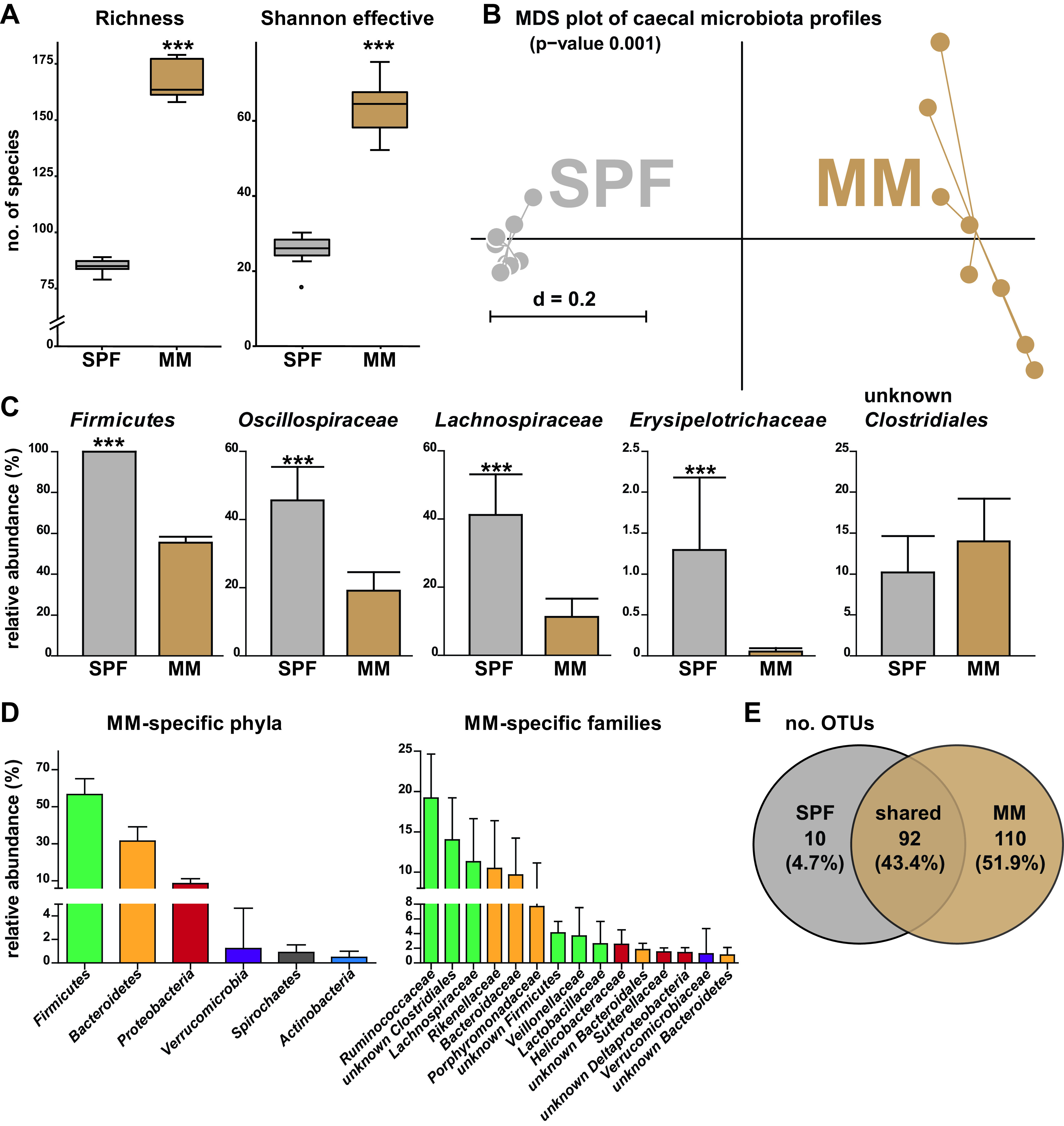
Microbiota profiles by 16S rRNA gene amplicon sequencing (*n *= 8 per group). All readouts were generated at the age of 58 days. Chickens treated with maternal microbiota (MM) after birth were compared to a control group kept under specific pathogen-free conditions (SPF). (A) Alpha diversity shown as species richness and Shannon effective counts. (B) Beta diversity shown as multidimensional scaling (MDS) plot based on generalized UniFrac distances. The *P* value was calculated by PERMANOVA. Stress value: 8.36e−05. (C) Microbial composition at the phylum and family level. Adjusted *P* values were calculated by Fishers exact test (*Erysipelotrichaceae*) or the Wilcoxon rank sum test (all other taxa): **, *P* < 0.01; ***, *P* < 0.001. (D) Cecal microbiota composition in the MM group at the phylum and family level. (E) Venn diagram showing unique and shared operational taxonomic units (OTUs) between the two colonization groups.

We then investigated the effects of the intervention on the gut microbiota via 16S rRNA gene amplicon analysis of cecal samples at day 58. Paired-end sequencing generated 116,040 high-quality, assembled reads (7,253 ± 2,562 reads/sample) representing a total of 212 operational taxonomic units (OTUs) (126 ± 43 OTUs/sample). Sequencing depth was sufficient, as indicated by all rarefaction curves reaching a plateau (Fig. S1 in the supplemental material). Alpha diversity of the microbiota was markedly higher in the MM versus SPF group (Fig. 2A). The phylogenetic makeup of communities also showed clearly distinct profiles, with pronounced interindividual differences in MM chickens (Fig. 2B).

Strikingly, *Firmicutes* was the only phylum detected in SPF animals, belonging primarily to the subordinate families *Oscillospiraceae*, *Lachnospiraceae*, *Erysipelotrichaceae*, and unknown members of the order *Clostridiales* (Fig. 2C). *Lactobacillaceae* and unknown representatives of the *Bacillales* were also observed in 37.5% of SPF samples (data not shown). In contrast, MM chickens were characterized by a more complex cecal microbiota composition, including members of multiple phyla: *Firmicutes*, *Bacteroidetes*, *Proteobacteria*, *Verrucomicrobia*, *Spirochaetes*, and *Actinobacteria* (Fig. 2D). The distribution of corresponding dominant families in MM chickens is shown in Fig. 2D. A search for unique and shared molecular species revealed 110 (51.9%) and 10 (4.7%) of 212 unique OTUs in the MM and SPF group, respectively, while 92 (43.4%) OTUs were shared (Fig. 2E).

In conclusion, these high-throughput sequencing data clearly show that immune system maturation in chickens exposed to maternal microbiota was associated with diverse gut microbial communities in the cecum.

### Cultivation revealed novel taxa and allowed functional studies.

To investigate the role of specific microbiota members in the effects of MM exposure as reported above, isolates were obtained by cultivation and a Chicken intestinal Bacterial Collection (ChiBAC) was created. The rationale was to cover a reasonable phylogenetic diversity at the species level, describe all collection members taxonomically, and make them publicly available. To this end, all isolates were deposited at the Leibniz Institute DSMZ-German Collection of Microorganisms and Cell Cultures and are accessible at www.dsmz.de/chibac.

**FIG 3:**
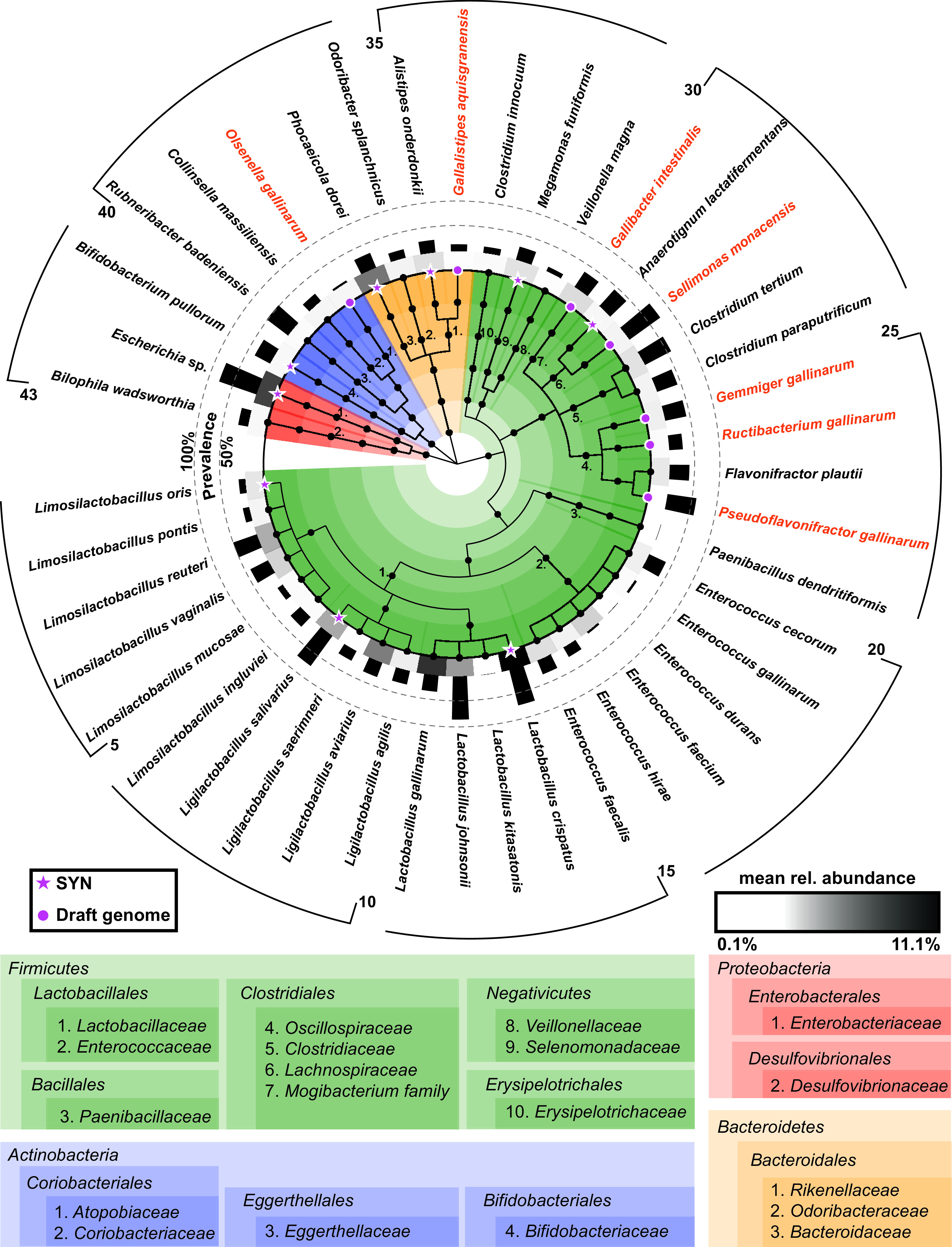
Diversity within the Chicken intestinal Bacterial Collection (ChiBAC). The cladogram shows all 43 isolates sorted according to their taxonomic classification. The colors correspond to phyla as indicated below the cladogram. Numbers indicate families within each phylum. Novel taxa described in the present study with their candidate names are indicated in orange. Isolates for which a draft genome was generated are indicated with a purple symbol (dots and stars). Stars indicate the strains selected to build the synthetic community (SYN). The gray scale in the inner circle depicts the average relative abundance of each isolate in 16S rRNA gene amplicon samples positive for the given species out of 1,499 chicken gut samples tested in total. Black bars in the inner circle next to the gray scales show the prevalence of each species, i.e., the number of positive samples.

From the 361 colonies picked and processed, 43 different species spanning 19 families across 4 phyla were kept in ChiBAC (Fig. 3 and Table S1 in the supplemental material). Most of these isolates belonged to the phylum *Firmicutes* (*n* = 33, 76.7%), especially the families *Lactobacillaceae* (*n* = 14, 32.6%) and *Enterococcaceae* (*n* = 6, 14.0%), followed by members of the phyla *Bacteroidetes* (*n* = 4, 9.3%), *Actinobacteria* (*n* = 4, 9.3%), and *Proteobacteria* (*n* = 2, 4.7%). The majority of these isolates was detected in publicly available 16S rRNA gene amplicon data sets (*n *= 1,499) (34), testifying to their prevalence in the chicken gut (Fig. 3 and Table S1). Of the 43 isolates, 7 represented potentially novel taxa, and the creation of 3 new genera and 4 new species is proposed to accommodate them. Draft genomes were generated and protologues describing each of these new bacteria and corresponding proposals for names are provided in the Materials and Methods section. Phylogenetic trees based on both 16S rRNA genes and draft genomes, along with electron micrographs of the respective bacterial cells, are available in Fig. S2.

### Synthetic bacterial community consisting of phylogenetically and functionally diverse bacteria.

A minimal bacterial consortium, hereon referred to as synthetic community (SYN), was then selected from the ChiBAC strains with the goal of modulating immune responses in hatched chickens. We restricted this selection to isolates with favorable growth behavior in the lab and belonging to risk group 1 taxa according to the TRBA 466 classification (Technical Rules for Biological Agents, Germany).

From the 16S rRNA gene amplicon profiles presented in Fig. 2, it became obvious that any members of phyla other than *Firmicutes* could be associated with the MM phenotype. Bifidobacterium pullorum (phylum *Actinobacteria*), Alistipes onderdonkii, and Phocaeicola dorei (phylum *Bacteroidetes*) were selected on this basis along with their phylogenetic diversity. Additionally, all three species were classified as abundant members of the chicken gut with average relative abundances of 0.4%, 5.4%, and 1.9%, respectively, according to 97% sequence identity matches to OTUs in MM chickens. Escherichia sp. strain DSM 109009 was selected as a member of one additional phylum (*Proteobacteria*) because of its facultative anaerobic growth behavior that may facilitate the establishment of strict anaerobes (35). The presence of flagella for this species was confirmed by negative staining (see the Materials and Methods section) (Fig. S3). Within the phylum *Firmicutes*, members of the dominant family *Lactobacillaceae*, including lactobacilli, but also members of the recently described genera *Ligilactobacillus* and *Limosilactobacillus* (36), and of the genus *Megamonas*, were underrepresented in SPF chickens, which justified the addition of Lactobacillus crispatus, Limosilactobacillus oris, Ligilactobacillus salivarius, and Megamonas funiformis to SYN. Finally, Anaerotignum lactatifermentans, a known butyrate producer within the family *Lachnospiraceae*, was added (37). Butyrate and isobutyrate production by the latter species was verified *in vitro* by high-performance liquid chromatography (HPLC).

**FIG 4:**
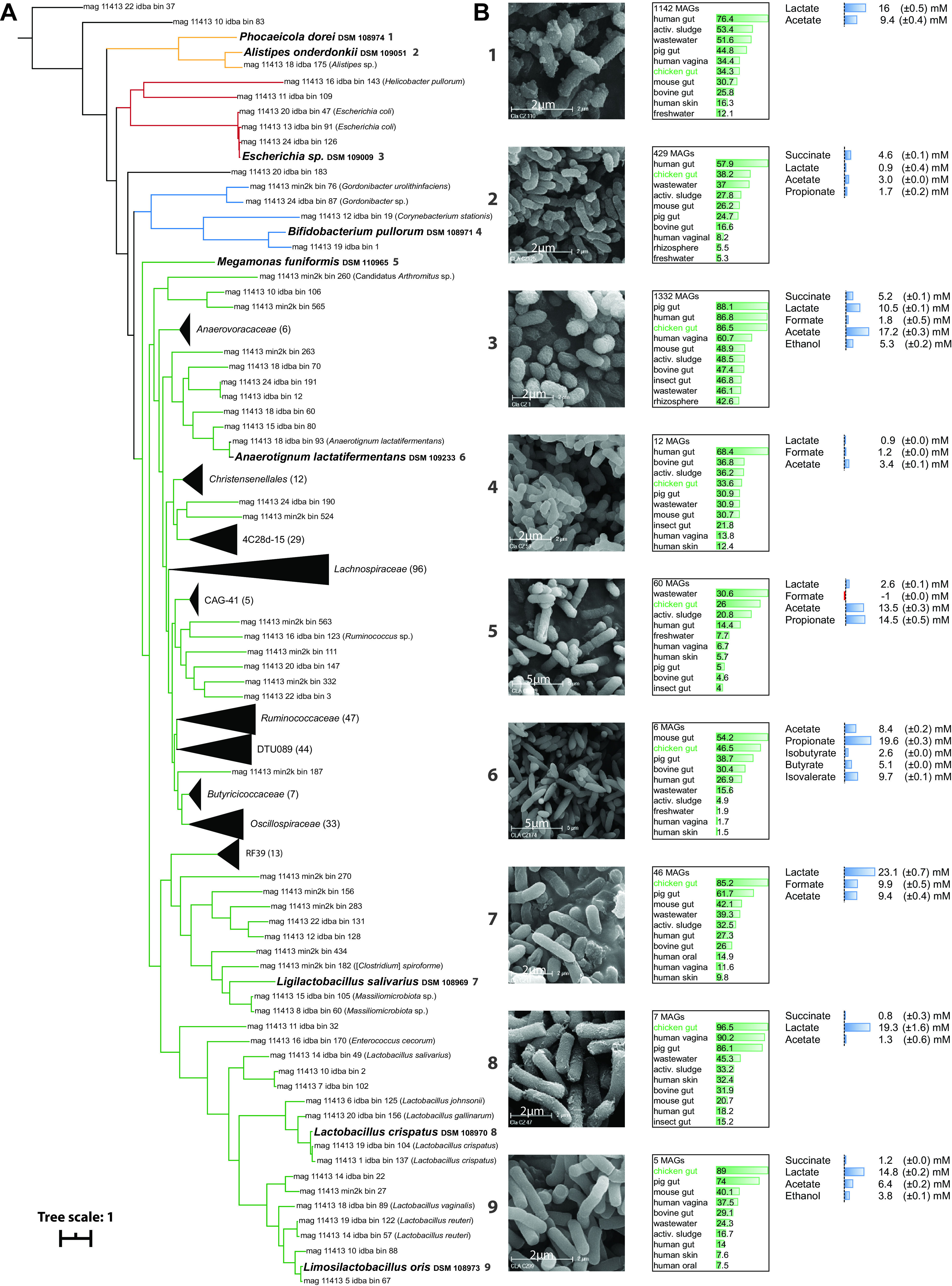
Diversity and main features of the nine SYN species. (A) Phylogenomic tree showing the placement of SYN members among a comprehensive, publicly available collection of metagenome-reconstructed genomes (MAGs) from the chicken gut (106). Whenever MAGs were collapsed into taxonomically congruent clusters (represented by black triangles) for the sake of clarity, their taxonomy and corresponding number are indicated in brackets. Species-level identification of MAGs is shown in brackets after their ID number whenever appropriate. All other MAGs correspond to yet unknown bacteria. Branches of the tree are colored by phyla as in Fig. 3. The SYN species are numbered from top to bottom and their properties are shown in the other figure panels. (B) Electron micrographs, sequence-based ecology, and metabolite profiles of each SYN member. Metabolite profiles were determined by HPLC-RI as described in the methods. For the ecological distribution, the percentages written in the bars indicate the fraction of IMNGS processed 16S rRNA gene amplicon data sets (*n *= 1,000) positive for the corresponding species (>97% sequence identity) in the given habitat. The 10 habitats with the highest prevalence are shown for each isolate. The number of MAGs matching the genome of the corresponding isolate (MASH distance of <0.05) out of a total of 52,605 MAGs (20, 24, 93–103) is also indicated.

Details about all nine SYN species, including genome-based phylogeny, electron micrographs, metabolite production, ecological distribution, and further information, such as their origin and the presence of antimicrobial resistance genes, are presented in Fig. 4 and Table S1.

### SYN partially colonized the cecum and influenced IgA levels transiently.

To investigate whether the selected species within SYN can affect Ig levels *in vivo* and colonize the gut of chickens, an intervention trial was performed as detailed in the methods section.

**FIG 5:**
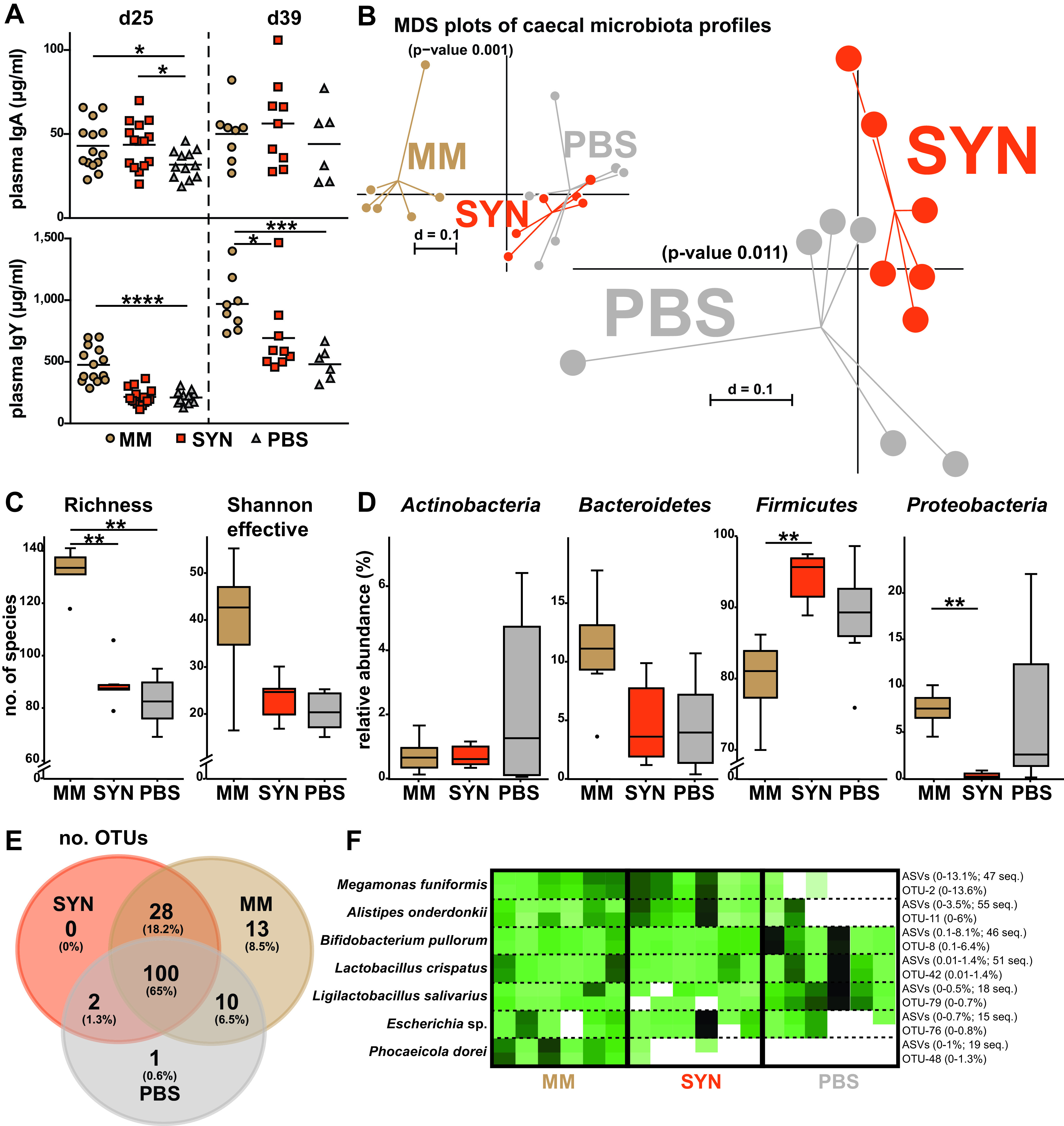
*In vivo* intervention trial with the synthetic bacterial community (SYN). (A) Immunoglobulin concentrations in plasma at day 25 (*n*_MM_ = 14, *n*_SYN_ = 15, *n*_PBS_ = 12) and day 39 (*n*_MM_ = 8, *n*_SYN_ = 9, *n*_PBS_ = 6) as measured by ELISA. (B) Microbial beta diversity in cecum at day 25 shown as multidimensional scaling (MDS) plots based on generalized UniFrac distances (*n *= 6 in all groups). The stress value for pairwise comparison SYN versus PBS = 0.128. (C) Alpha diversity shown as species richness and Shannon effective counts. (D) Microbial composition at the phylum level. (E) Venn diagram showing unique and shared operational taxonomic units (OTUs) between the colonization groups. (F) Heat map of SYN members using either an OTU- or amplicon sequence variant (ASV)-based approach for data analysis. The percentages in brackets indicate the range of relative abundances of the corresponding taxa across all samples. For ASVs, the number of sequences sharing >99% sequence identity with the SYN members is also indicated. Statistics for immunoglobulin measurement were performed with a Mann-Whitney U test. Statistics for microbial profiles were performed using a Wilcoxon rank sum test: *, *P* < 0.05; **, *P* < 0.01; ***, *P* < 0.001; ****, *P* < 0.0001.

At day 25 of age, plasma IgA, but not IgY levels, were significantly higher in chickens that received SYN treatment compared with birds receiving phosphate-buffered saline (PBS; negative control), reaching an average level comparable to MM positive-control animals (Fig. 5A) (*P* < 0.05; Mann-Whitney U test). However, this difference in IgA did not prevail after 39 days, while IgY levels in SYN chickens were intermediate between the placebo and MM control (*P* = 0.0152) groups at this second time point measured.

Cecal samples were analyzed at day 25, the time point at which differences were observed for the main study readout IgA, by means of 16S rRNA gene amplicon sequencing to assess microbiota profiles and detect colonization by the SYN members. A total of 269,194 high-quality reads (14,955 ± 5,875 reads/sample) representing 156 OTUs (101 ± 24 OTUs/sample) were included in the analysis. Sequencing depth was sufficient, as indicated by all rarefaction curves reaching a plateau (Fig. S1). Beta diversity revealed a distinct clustering of MM chickens and a slight effect of SYN on the overall phylogenetic makeup of cecal microbial communities compared with the PBS group (Fig. 5B). A similar trend of MM being distinct to the two other groups was observed for alpha-diversity parameters (Fig. 5C) and phylum composition (Fig. 5D). At the resolution of single molecular species (Fig. 5E), the SYN group shared more OTUs with MM chickens than the control PBS group (18.2% versus 6.5%), while all three groups shared the majority of OTUs (65% in common).

We then specifically searched for OTUs matching the reference 16S rRNA gene sequences of SYN members at an identity of >97%. For a better resolution, the same data were reanalyzed at the level of amplicon sequence variants (ASVs) using the DADA2 pipeline (38) and the ASVs were matched to the reference sequences at a threshold of >99% identity. The results of both analyses were consistent and are displayed in Fig. 5F. Sequence hits were detected for seven of nine SYN members; sequences corresponding to Limosilactobacillus oris and Anaerotignum lactatifermentans were absent from all samples. The occurrence of both Megamonas funiformis and Alistipes onderdonkii was higher in SYN versus PBS chickens (*P* = 0.007 and 0.030, respectively; Mann-Whitney U test), reaching relative abundances comparable to MM controls or even higher (maximum 13.6% and 6% relative abundance, respectively). Escherichia sp. and Phocaeicola dorei were detected in two additional SYN animals compared to PBS controls, albeit at relatively low relative abundances. Bifidobacterium pullorum, Lactobacillus crispatus, and Ligilactobacillus salivarius were present in all three groups of chickens, the latter species being in low abundance. Altogether, these data revealed marked interindividual differences and suggested successful colonization for two of nine SYN member strains.

## DISCUSSION

Although chicken are very important domestic animals, knowledge about their gut microbial communities is still scant compared to mice and humans. We thus studied chicken gut bacteria and their interactions with the immune system via a combination of cultivation and sequencing techniques. This opens new avenues of research and applications to tackle issues of relevance for the environment and animal wellbeing.

We first demonstrated the stimulating effects of complex fecal communities on the developing immune system in chickens. Antibody-related gene transcripts were upregulated and antibody levels were significantly higher in treated chickens. Separating chickens geographically and keeping them under different hygienic standards may have affected their gut microbiome besides fecal microbiota uptake (39). Nonetheless, we observed a clearly different immunological phenotype associated with a more diverse microbiota structure.

The concept of giving intestinal content to newly hatched chicks was established by Nurmi et al. to prevent Salmonella enterica serovar Infantis infection (25). Already in 1973, they stated that “broiler production is carried out under abnormally hygienic conditions” and proposed that intestinal microbiota will normally build defense mechanisms against pathogens (25). Goren et al. (26) and Varmuzova et al. (13) further developed this principle, and both groups focused on competitive exclusion against Salmonella.

The latter study also demonstrated higher gene expression of IgA and IgY in cecal tissue of chickens treated with donor microbiota from 42-week old adult birds, an effect that supports our own qPCR and ELISA results. Inoculation of hatched chickens with fecal microbiota was also carried out by Volf et al. to detect differences in gene expression in cecal tissue (40). Significantly lower expression of AVD, IgM, Ig lambda, CALB1, ES1, and ISG12-2 were identified in germ-free compared to colonized chickens (40). A study published in 2017 compared germ-free chickens to mono-colonization with either Escherichia coli strain Nissle 1917 or Enterococcus faecium strain DSM 7134, or to tetra-colonization with Escherichia coli Nissle 1917, Enterococcus faecium DSM 7134, Lacticaseibacillus rhamnosus strain DSM 7133, and Clostridium butyricum strain DSM 10702^T^, and to conventional chickens (with a complex microbiome) by gene expression of immunoglobulins. The tetra-colonized group showed IgA and Ig lambda expression levels comparable to conventional animals, while the effect of mono-colonization was weak. Ig transcripts were not detected in germ-free animals (41).

Oral inoculation at hatch with the aforementioned tetra-colonization mixture also partially protected chickens against Salmonella enterica challenge, while colonization with adult hen microbiota entirely prevented Salmonella infection (42). In contrast to these published studies, we used an experimental setting mimicking native conditions with complex colonization instead of germ-free controls and combined host and microbiome-related approaches to test causation.

To dissect molecular mechanisms underlying microbe-host interactions, it is crucial to have access to well-described isolates to perform mechanistic studies. Useful collections of bacterial isolates have been generated for humans (21, 43–45), mice (46, 47), cows (22), and pigs (24). While this work was ongoing, a collection of 133 chicken gut anaerobes was published by Medvecky et al. (19); however, the strains are not publicly available. Thus, we made the effort to create a repository of publicly available isolates representing 43 species, including seven novel bacteria that were described taxonomically. Based on this cultivation work, a synthetic bacterial community (SYN) consisting of nine bacterial isolates was designed and used *in vivo*. Promising results were obtained with respect to boosting systemic IgA levels, but this effect was only transient (observed at day 25 but not at day 39 of age). Even though a long-lasting effect was not observed, elevated IgA levels at the earlier time point in the SYN group may still provide superior immunity in a phase where chicks are susceptible to pathogens.

Of note, the gut microbiota of PBS control chickens in this intervention trial was much more diverse than the SPF control animals examined in the first trial, with which we studied the impact of maternal microbiota colonization. The high diversity of naturally occurring species in the PBS control chickens may have partly contributed to the limited engraftment of SYN members, i.e., due to the presence of a complex microbiota within these animals, the niches for each of the SYN species may already have been occupied by other members of the microbiota. Refinements of the procedure to prepare SYN working stocks for colonization will also be required in the future. For instance, freezing may have potentially damaged cells and the effects of a freshly grown mixture will be worth investigating (48–50). The exposure to oxygen (up to 5 min) before gavaging birds was possibly detrimental (51). Next, considering the hostile environment of chicken stomachs (pH <3.5), losses may have occurred during transition of SYN species, as described for ingested bacterial strains in humans (52). Moreover, in the gut of mammals, it is known that the microbiota develops from an aerobic/facultative anaerobic to an obligate anaerobic state; thus, even though gavaging was repeated three times here, it may be useful to increase the frequency of intervention to ensure engraftment of strictly anaerobic species (35). Kubasova et al. recently found that members of the phylum *Firmicutes* tend not to colonize the intestine of chicken, with the exception of members of the class *Negativicutes* (53). This is contradictory to other results from the literature reporting that *Lachnospiraceae* and *Oscillospiraceae* were found in 3- or 4-day-old broiler and layer chickens, albeit without bacterial intervention (54), but corroborates our own findings that Anaerotignum lactatifermentans and *Limosilactobacillus oris* were not detected after SYN intervention, and other lactobacilli were only present in low relative abundances.

In summary, the first steps were taken toward the development of synthetic bacterial communities as starter microbiota for newly hatched chickens. Further studies will be needed to expand the array of cultured gut bacteria available from chicken and to optimize colonization strategies based on synthetic communities.

## MATERIALS AND METHODS

### Animal trials.

All experiments were ethically approved by either (i) the French Ministry of Education and Research (Ministère de l’éducation nationale, de l’enseignement supérieur et de la recherche) under protocol number APAFIS 5833-20l60624l6362298 v3, or (ii) the government of Upper Bavaria (Regierung von Oberbayern), Department 54, veterinary affairs, under protocol number 55.2-1-54-2532.0-60-2015.

### Maternal microbiota study.

Chickens of the INRAE PA-12 line, an outbred white Leghorn line with heterozygous MHC haplotype B21, were used. All eggs originated from the animal facility at INRAE in Tours, France. Eggs were either (i) allowed to hatch in the same facility and kept under specific pathogen-free (SPF) conditions (*n *= 17) or (ii) shipped for subsequent intervention with maternal microbiota (MM; *n *= 25). Concerning the former, SPF rooms had to be entered with gloves and overalls through disinfection pads. Monitoring included the absence of Salmonella enterica serovar Gallinarum biovar Pullorum, Mycoplasmoides gallisepticum, Mycoplasmopsis synoviae, layer fall syndrome, Adenovirus, Newcastle disease, infectious bursal disease, influenza, infectious bronchitis, infectious laryngo-tracheitis, and Mareks’ disease. Animals were regularly examined and showed no signs of infection. The facility was checked regularly for Salmonella contaminations. Concerning chicken for MM trials, eggs were transported to the Institute of Animal Physiology at the Ludwig-Maximilians-University (LMU) in Munich where they were subjected to the intervention with maternal microbiota. Toward this end, five adult Lohmann selected Leghorn (LSL) chickens were hosted in the aviary in which they were allowed to defecate during the 3 days preceding hatch. Hatched chickens were then placed into this aviary for passive colonization. No specific hygienic measures were applied for MM chickens. All animals were culled at day 58 post hatch. Heparin blood samples were collected immediately before culling, centrifuged (1,500 × *g*, 10 min) and plasma was stored at −20°C. Bile was collected from the gallbladder and frozen at −20°C (this failed for two MM chickens). Cecal contents (*n *= 8 in both groups) were immediately frozen at −80°C. Cecal tonsils (*n *= 6 in both groups) were collected in RNAlater (Merck KGaA, Darmstadt, Germany) and kept at 4°C for 24 h prior to freezing at −80°C.

### Targeted colonization study with the synthetic community.

This animal experiment was conducted at the Institute of Animal Physiology of the LMU using chickens of the INRAE PA-12 line provided from the animal facility at INRAE in Tours, France. Hatched chickens were equally split into three groups and immediately brought to the experimental animal facility. Before the animals were assigned to their respective aviaries, the synthetic bacterial community (SYN) and PBS control group received 250 μl of SYN (see details below) or sterile PBS, respectively, by gavaging directly into the crop with a button cannula. This procedure was repeated on day 1 and 2 post hatching. For the MM group, the aviary was spread with feces freshly collected from adult LSL layer chickens (500 g initially and then 200 g on day 1 and 2 post hatching). Heparin blood samples were collected at day 25 and day 39, centrifuged (1,500 × *g*, 10 min), and plasma was stored at −20°C. At day 25, six animals in each group were culled and cecal contents were collected and stored in Stool DNA Stabilizer (Invitek Molecular GmbH, Berlin, Germany) at −80°C.

### Bulk RNA sequencing.

Cecal tonsils from six chickens in each of the SPF and MM groups (trial 1) at day 58 were homogenized in a Precellys homogenizer (VWR International GmbH, Darmstadt, Germany) (6,500 × *g*, 30 s). RNA was isolated using the peqGOLD TRIfast (VWR International GmbH, Darmstadt, Germany) reagent according to the manufacturer’s instructions. Concentrations and quality were measured using a NanoDrop1000 (Thermo Fisher Scientific, Waltham, MA, USA) and a 2100 Bioanalyzer (Agilent Technologies, Santa Clara, CA, USA). After quality control, only RNA samples with an RNA integrity number (RIN) of ≥8 were considered. Four SPF and six MM samples met the criteria for RNA sequencing, which was performed at the Center for Translational Cancer Research (TranslaTUM) of the Technical University of Munich. Libraries for bulk 3′-sequencing of poly(A)-RNA were prepared as described by Parekh et al. (55). Briefly, barcoded cDNA of each sample was generated with a Maxima RT polymerase (Thermo Fisher Scientific, Waltham, MA, USA) using oligo-dT primer containing barcodes, unique molecular identifiers (UMIs), and an adapter. The 5′ ends of the cDNAs were extended by a template switch oligonucleotide (TSO) and, after pooling of all samples, full-length cDNA was amplified with primers binding to the TSO-site and the adapter. The cDNA was fragmented and TruSeq-Adapters ligated with the NEBNextR Ultra II FS DNA library prep kit (New England BioLabs, Frankfurt am Main, Germany) for Illumina and 3′-end fragments were finally amplified using primers with Illumina P5 and P7 overhangs. In contrast to Parekh et al. (55), the P5 and P7 sites were exchanged to allow sequencing of the cDNA in read 1 and barcodes and UMIs in read 2 to achieve a better cluster recognition. The library was sequenced on a NextSeq 500 (Illumina, San Diego, CA, USA) with 65 cycles for the cDNA in read 1 and 16 cycles for the barcodes and UMIs in read 2. Data were processed using the published Drop-seq pipeline (v1.0) to generate sample- and gene-wise UMI tables (56). The reference chicken genome GRCg6a (Gen Bank accession GCA_000002315.5) was used for alignment. Transcript and gene definitions were used according to the ENSEMBL annotation release 98. The “differences in gene expression” (DGE) matrix was further analyzed in DEBrowser (57). Filtering (exclusion of genes with ≤2 reads/sample on average), median ratio normalization (MRN), DGE algorithms and statistical tests (Wald-Test, Benjamini and Hochberg [58]) were applied by DEseq2 (59, 60), including a significance cutoff (adjusted *P* value of <0.01) and a fold change cutoff (FC) of ≥2.

### Quantitative RT-PCR.

The same RNA samples as used for RNA sequencing (*n *= 6 per group; RIN ≥6.5; 1 μg each) were treated by DNase I (Thermo Fisher Scientific, Waltham, MA, USA) for 30 min at 37°C. EDTA (2.5 mM) was added and the samples were incubated for 10 min at 65°C to inactivate the DNase I. The Promega GOscript reverse transcriptase kit (Promega, Madison, WI, USA) was used to transcribe 400 ng of RNA into cDNA according to the manufacturer’s instructions. The cDNA was used as the template for SYBR Green-based quantitative RT-PCR with the GoTaq qPCR MasterMix (Promega, Madison, WI, USA) according to the manufacturer’s instructions. The 18S rRNA genes were used as housekeeping genes (F-5′ CAT GTC TAA GTA CAC ACG GGC GGT A; R-5′ GGC GCT CGT CGG CAT GTA TTA). The genes for IgA (F-5′ CGC CCC TTC CGT CTA CGT; R-5‘CGA AAT CGG TTG GTT TTG TTG), AID (F-5‘CGT CTG AAA CCC AGC AAG AGT; R-5′ TGT CCA TGT CAG CTG GGT TCT), and IL-6 (F-5′ GCT TCG ACG AGG AGA AAT GC; R-5′ GCC AGG TGC TTT GTG CTG TA) were amplified with annealing at 59°C. The genes for IgY (F-5′ TGG AGG GAA GGG AAG AGT TAC AG; R-5‘TCC GGG CAT CCC TTG AC), IgJ (Qiagen QuantiTect Primer Assay, Gg_IGJ_1_SG), and IL-21 (Qiagen QuantiTect Primer Assay, Gg_IL-21_1_SG) (Qiagen, Hilden, Germany) were amplified with annealing at 56°C. Cycling conditions were 2 min at 95°C followed by 40 amplification cycles (15 s at 95°C, 30 s at the specific annealing temperature, 30 s at 72°C). Specific PCR products were confirmed via melting curves. Nontemplate controls were included in each assay. qPCR was performed on a 7300 Real-Time PCR System (Applied Biosystems, Foster City, CA, USA). 18S rRNA gene expression was used for normalization (= dCT) and relative expression was calculated for each gene (= 40-dCT).

### Enzyme-linked immunosorbent assays.

Immunoglobulins (IgA and IgY) were quantified in samples from both animal trials by enzyme-linked immunosorbent assay (ELISA) according to Kothlow et al. (61). Briefly, 96-well plates were coated overnight with 2 μg/ml or 2.5 μg/ml murine monoclonal antibodies (mABs) in coating buffer (pH 9.6) for IgA (A1, SouthernBiotech, Birmingham, AL, USA) and IgY (G1, SouthernBiotech, Birmingham, AL, USA), respectively. Plates were blocked in 1% casein (Merck KGaA, Darmstadt, Germany) in PBS (pH 7.4) followed by subsequent incubations at room temperature with serial dilutions of chicken plasma, bile, or cecal content samples. Horseradish peroxidase (HRP)-coupled mABs against chicken IgA (A3-HRP) and IgY (G1-HRP) were used at a concentration of 1:10,000 in 1% casein in PBS-T (pH 7.4) followed by detection with tetramethylbenzidine (Merck KGaA, Darmstadt, Germany). Gibco chicken serum (Thermo Fisher Scientific, Waltham, MA, USA) was used as a standard control (IgA: 81 μg/ml; IgY: 1,685 μg/ml) to quantify immunoglobulins.

### 16S rRNA gene amplicon sequencing and analysis.

Cecal samples were processed according to Just et al. (62). Briefly, following mechanical cell lysis, metagenomic DNA was purified on columns (Macherey-Nagel, Düren, Germany). The V3/V4 regions of 16S rRNA genes were amplified (25 cycles in total) via a two-step PCR using primers 341F and 785R (63) following a combinatorial, dual-barcoding strategy. Amplicon libraries were sequenced in paired-end mode on a MiSeq (Illumina, San Diego, CA, USA) following the manufacturer’s instructions. Raw sequence reads were processed using IMNGS (34), a platform based on UPARSE (64). The parameters were as follows: barcode mismatches, 2; expected error, 2% (MM trial) or 3% (SYN trial) of sequence length; quality trimming score, 20 (MM trial) or 3 (SYN trial); trimming length, 20 nucleotides (nt) (MM trial) or 10 nt (SYN trial); minimum sequence length, 300 nt (MM trial) or 350 nt (SYN trial); maximum sequence length, 600 nt (MM trial) or 650 nt (SYN trial). Operational taxonomic units (OTUs) were clustered at 97% sequence similarity, including only those occurring at ≥0.25% relative abundance in at least one sample. Data sets were further analyzed using Rhea (65), a modular pipeline for microbial profiling of 16S rRNA gene amplicon sequencing data and effective diversity measures were calculated according to Jost (66, 67). To evaluate colonization efficiency of SYN members, the OTU table was prescreened using BLASTN (v2.6.0+) (68) at an identity threshold of 97% to reference sequences of the SYN bacteria. The identity of matches was confirmed using EZBiocloud (www.ezbiocloud.net) (69). Moreover, data were also analyzed using an ASV-based approach in DADA2 (v1.12.1) (38) with the recommended settings for paired-end sequences (adjusted options: maxEE, 3.3; truncQ, 2; maxN, 0; truncLen, [250, 250]). Error prediction was conducted using the pooled data. ASV sequences were assigned to SYN members using USEARCH (v8.1) (70) (query coverage, 80%; E value, 1e−25; identity, >99%).

### Gut sample collection for bacterial cultivation.

LSL M11 chickens housed at the Institute of Animal Physiology, LMU, were used as donors. Cecal, jejunal, and colonic contents were collected from three 7-day-old, five 21-day-old, and five 35-day-old animals. Four samples were taken from adult animals that were approximately 6 months old. Additionally, cecal, jejunal, and colonic samples from a free-range layer and a barn layer from Dietramszell, Germany, and a barn broiler from Geretsried, Germany, were collected to increase the starting pool of bacterial diversity for isolation.

Prior to culling animals, Schott bottles containing 80 ml of PBS or Wilkins-Chalgren-anaerobe (WCA) (Thermo Fisher Scientific, Waltham, MA, USA) broth were gassed (5.7% CO_2_, 4.7% H_2_, 89.6% N_2_) for approximately 10 min to enable anoxic conditions and were subsequently autoclaved (121°C, 15 min). Intestinal content was first suspended in anoxic PBS (ca. 10-fold dilution; wt/vol). After 2 min of vigorous shaking, mixtures were left to stand for approximately 1 min to sediment debris and 5 ml of the slurry was transferred to the anoxic WCA medium using a sterile syringe fitted with a needle. Samples were brought to the lab (maximum of 1 h of transportation) for cryo-conservation by mixing the slurry 1:1 with sterile and anoxic WCA medium containing 40% glycerol. Samples were stored at −80°C until further processing.

### Culture media.

The culture medium used for the isolation of each strain is listed in Table S1 in the supplemental material. All media were supplemented with a redox potential indicator (phenosafranine, 2.5 mg/liter) and reducing agents (dithiothreitol, 0.2 g/liter; l-cysteine, 0.5 g/liter). Agar (15 g/liter) was added if solid medium was needed. To enhance the growth of fastidious bacteria, some media were supplemented with 5% sheep blood (ILMED Labor- und Medizintechnik GmbH, Germany) whenever indicated in Table S1. The following media were prepared as indicated by the manufacturer: brain heart infusion (BHI) (Thermo Fisher Scientific, Waltham, MA, USA); Gifu anaerobic medium (GAM) (HyServe GmbH, Uffing, Germany); Gifu anaerobic medium, modified, (GAM-mod) (HyServe GmbH, Uffing, Germany); Wilkins-Chalgren-anaerobe broth, WCA (Thermo Fisher Scientific, Waltham, MA, USA). Yeast extract, casitone, and fatty acid (YCFA) medium was prepared for additional HPLC-RI measurements according to the instructions of the DSMZ (DSMZ Medium 1611, https://www.dsmz.de/microorganisms/medium/pdf/DSMZ_Medium1611.pdf), but it was gassed with a different gas mixture (5.7% CO_2_, 4.7% H_2_, 89.6% N_2_).

### Bacterial cultivation, isolation, and storage.

Frozen samples were brought into an MBraun anaerobic workstation (M. BRAUN INERTGAS-SYSTEME GmbH, Garching, Germany) containing a gas mixture of 95% CO_2_, 4.75% N_2_, and 0.25% H_2_. Serial dilutions of samples (10^−2^ to 10^−5^) in PBS were plated on solid culture medium. Plates were incubated at 37°C and checked daily for the occurrence of new colonies. Single colonies were streaked at least three times to guarantee purity and then transferred into liquid medium using the Hungate technique (71) and following the instructions of the DSMZ for cultivation of strictly anaerobic bacteria (72). Hungate tubes were incubated at 37°C until turbidity was visible. Liquid cultures were further used for identification by matrix-assisted laser desorption ionization (MALDI) biotyping and sequencing and for cryo-conservation of single isolates as described above for gut samples.

### Strain identification.

A MALDI-Biotyper (Bruker, Billerica, MA, USA) was used for initial identification of cultures. Liquid cultures were centrifuged (12,000 × *g*, 10 min), prepared as described elsewhere (73), and used as biomass for identification following the manufacturer’s instructions. The identity of all isolates included in the final collection was confirmed by amplification of the 16S rRNA gene using primers 27F (5′ AGA GTT TGA TCA TGG CTC A 3′) and 1492F (5′ TAC GGT TAC CTT GTT ACG ACT T 3′). After PCR product cleanup, Sanger sequencing was performed using primers 27F, 338R (5′ GCT GCC TCC CGT AGG AGT 3′), 785F (5′ GGA TTA GAT ACC CTG GTA GTC 3′), and 1492R. Contigs were built in MEGA v7 (74) and the full-length sequences were searched for closely related species with a valid name using EZBiocloud (69). A 16S rRNA gene sequence identity of ≤98.7% was considered the threshold for novel species delineation (75) and a draft genome was generated for all corresponding isolates.

### Preparation of cryo-aliquots of the synthetic bacterial community (SYN).

One cryo-aliquot (300 μl) of each SYN strain (see Results section) was used to inoculate individual Hungate tubes filled with 9 ml of WCA broth prior to growth at 37°C for 1 day. Cultures were subcultured once and incubated at 37°C for 2 days before they were brought into a Whitley A35 HEPA anaerobic workstation (Don Whitley Scientific Limited, Bingley, UK) and mixed at equal volumes. Cryo-stocks were prepared by mixing the SYN suspension with the same volume of sterile WCA medium supplemented with 40% glycerol as cryo-protectant. SYN cryo-stocks (3 ml) were distributed into 5 ml tubes, frozen on dry ice, and stored at −80°C until the start of the intervention trial.

### Electron microscopy.

For scanning electron microscopy (SEM), cryo-aliquots (300 μl) were used for inoculating Hungate tubes filled with 9 ml of modified BHI medium (DSMZ medium 215c), followed by incubation at 37°C until a dense growth was observed (1 to 3 days). Next, 1.5 ml of culture was centrifuged (12,000 × *g*, 10 min) and the supernatant was discarded. Cells were fixed with 3% (vol/vol) glutaraldehyde (Agar Scientific, Stansted, UK) in 0.1 M Sorensen’s phosphate buffer, washed in phosphate buffer for 15 min, and dehydrated by incubating consecutively in an ascending ethanol series (30%, 50%, 70%, 90%, and 100%) for 10 min each and the last step thrice. The samples were critical point dried in liquid CO_2_ (Polaron, GaLa Instrumente, Bad Schwalbach, Germany) and sputter coated (Sputter Coater EM SCD500; Leica, Wetzlar, Germany) with a 10 nm gold/palladium layer. Samples were analyzed using an environmental scanning electron microscope (ESEM XL30 FEG, FEI, Eindhoven, Netherlands) with a 10 kV acceleration voltage in a high-vacuum environment.

Negative staining was applied for Escherichia sp. DSM 109009 to visualize flagella. Therefore, fresh biomass in growth medium was allowed to adsorb on Formvar-carbon-coated nickel grids (Maxtaform, 200 mesh, Plano, Wetzlar, Germany) for 10 min. Grids were washed with distilled water. Samples were stained by placing a drop of 1% phosphotungstic acid (in distilled water [pH 7.2], Agar Scientific, Stansted, UK) on the grid for a few seconds. The grid edge was carefully laid to filter paper to remove the adhesive drop from the grid. After air drying, samples were examined using a Hitachi HT7800 transmission electron microscope (Hitachi, Japan) operating at an acceleration voltage of 100 kV.

### Short-chain fatty acid production.

For the nine SYN isolates, the production of short-chain fatty acids (SCFA) (acetate, propionate, butyrate, valerate), branched SCFAs (isobutyrate, isovalerate), and intermediate metabolites (lactate, succinate, formate), as well as ethanol, were determined using high-performance liquid chromatography with refractive index detection (HPLC-RI). Bacteria were grown in BHI broth or YCFA broth supplemented with 0.02% (wt/vol) dithiothreitol (DTT) and 0.05% (wt/vol) l-cysteine (0.1% [wt/vol] in YCFA) in Hungate tubes for 24 h at 37°C under anaerobic conditions and with shaking (200 rpm). Triplicate cultures were measured for each strain. Negative controls consisted of medium without bacteria. After incubation, samples were centrifuged (10,000 × *g*, 10 min, 4°C) and supernatants were filtered into 2-ml short thread vials with screw caps (VWR International GmbH, Darmstadt, Germany) using nonsterile 0.2-μm regenerated cellulose membrane filters (Phenomenex, Aschaffenburg, Germany) and stored at −20°C until measurement. Samples were measured with a Hitachi Chromaster 5450 (VWR International GmbH, Darmstadt, Germany) fitted with a refractive index detector and a Shodex SUGAR SH1011 column (300 × 8.0 mm) (Showa Denko Europe, Munich, Germany). A Shodex SUGAR SH-G (6.0 × 50 mm) was used as guard column. The injection volume was 40 μl. The running temperature was 40°C. The eluent was 10 mM H_2_SO_4_ with a constant flow of 0.6 ml/min. Concentrations were determined using external standards via comparison of the retention time (all compounds were purchased from Sigma-Aldrich). Peaks were integrated using the Chromaster System Manager software (Version 2.0, Hitachi High-Tech Science Corporation, Japan). The limit of quantification was set to an S/N ratio of 10.

### Cellular fatty acid analysis.

Cellular fatty acids of the novel isolates were analyzed after conversion into fatty acid methyl esters (FAMEs) by saponification, methylation, and extraction according to the method of Miller (76) and Kuykendall et al. (77), with minor adaptions. The FAME mixtures were separated by gas chromatography. They were detected by a flame ionization detector using the Sherlock Microbial Identification System (MIS) (MIDI, Microbial ID, Newark, DE, USA). Peaks were automatically integrated, and fatty acid names, as well as the percentages, were calculated by the MIS Standard Software (Microbial ID) referring to the ANAEROBE database. For the confirmation of the identity to resolve summed features of the MIDI analysis, a gas chromatography mass spectrometry (GC-MS) run was performed on a GC-MS 7000D (HP-5 ms UI 30 m × 250 μm × 0.25 μm column, helium flow 1.2 ml min^−1^, injection of 1 μl, split ratio of 7.5:1) (Agilent Technologies, Santa Clara, CA, USA). The following oven program was used: initial temperature 170°C, ramp 3°C/min to 200°C, ramp 5°C/min to 270°C, ramp 120°C/min to 300°C, and hold for 2 min. The inlet temperature was set to 170°C and then linearly increased with 200°C/min up to 350°C and held for 5 min. The mass spectrometry parameters were set to aux temperature 230°C, source temperature 230°C, and electron impact ionization at 70 eV with mass range of *m/z* 40 to 600. The identification of the peaks was performed based on retention time and mass spectra. The positions of single and double bonds were confirmed by a derivatization to the corresponding dimethyl disulfide adduct (78).

### Genome sequencing and assembly.

Draft genomes were generated for all isolates representing potentially novel taxa (*n *= 7) and for those included in SYN (*n *= 9). DNA libraries were prepared using the NEBNext Ultra II DNA library prep kit (New England BioLabs, Frankfurt am Main, Germany). Libraries were sequenced using the Illumina MiSeq (Illumina, San Diego, CA, USA) system according to the manufacturer´s instructions. Illumina reads were assembled using Spades (v.3.6.1) (79) with the activated BayesHammer tool for error correction and a MismatchCorrector module for post-assembly mismatch and indel corrections. Assemblies were evaluated using checkM (v.1.0.12) (80).

### Description of novel taxa.

Functional annotation required extraction of both coding DNA sequences (CDS) and clustered regularly interspaced short palindromic repeats (CRISPR) via Prokka (v1.14) (81). Protein sequences were checked against the Comprehensive Antibiotic Resistance Database (CARD) (downloaded January 2020) (82) and for carbohydrate activate enzymes (CAZymes) (downloaded July 2019) (83) using DIAMOND (>40% identity, >40% query and subject coverage) (84). The Prokka annotations were mapped to KEGG (85) using PROKKA2KEGG (https://github.com/SilentGene/Bio-py/tree/master/prokka2kegg).

Taxonomic assignment was based on both 16S rRNA gene and genome sequences. Each isolate’s 16S rRNA gene sequence was compared to the Living Tree Project (LTP) (v132) (86) and pairwise sequence identities were calculated (69). The 50 best matches with a valid name and for which a genome exists were compared to the isolate using both average nucleotide identity (ANI) via FastANI (v1.3) (87) and the percentage of conserved proteins (POCP) (88). Phylogenomic trees were created using the genomes of the input isolate and its closest relatives with PhyloPhlan3 (v3.0.53) (89). For confirmation of species delineation, the genome of target isolates was entered into the type-strain genome server (TYGS) to determine digital DNA:DNA hybridization (dDDH) values (90).

For taxa delineation, 16S rRNA gene sequence identities of ≤98.7% and ≤94.5% were indicative for novel species and genera, respectively (75). ANI values of <95% between genomes were considered an indication for separate species. If ANI values were close to 95% or inconsistent with the 16S rRNA assignment, dDDH was consulted with a species delineation value of ≤70%. A difference in G+C content of genomic DNA ≥1% was considered a further indication of species-level differentiation (91). POCP values of ≤50% were indicative for the creation of a new genus (88). The classification of isolates was further confirmed using GTDB-Tk (v1.2.0) (92) and by constructing 16S rRNA gene-based and phylogenomic trees (Fig. S2). A database of publicly available MAG data sets (20, 24, 93–102) was compared to isolates using MASH (v2.2) (103). Only those with a distance score of <0.05 were reported. All taxonomic and functional data used to create the protologues mentioned below are available at https://github.com/thh32/Protologger within the data sets section.

**(i) Description of *Gallibacter* gen. nov.** *Gallibacter* (Gal.li.bac’ter. L. masc. n. *gallus*, chicken; N.L. masc. n. *bacter*, rod; N.L. masc. n. *Gallibacter*, a chicken rod). The genus falls into the family *Eubacteriaceae* (phylum *Firmicutes*) according to 16S rRNA gene-based phylogeny (Fig. S2A). The closest relatives are Eubacterium brachy (93.3% sequence identity), Eubacterium infirmum, and Anaerovorax odorimutans (both 90.7%). POCP analysis suggests the input genome to belong to the genus *Eubacterium* with a matching value of 54.8% to Eubacterium brachy. However, the type species of this genus Eubacterium limosum, shared a POCP value of 22.5% with the isolate. GTDB-Tk assigned the isolate to the family “*Anaerovoraceae”* (not valid), genus *Eubacterium*_M, showing that the current taxonomic status of the genus *Eubacterium* is incongruent. Therefore, a novel genus with the name *Gallibacter* is proposed to accommodate the isolate. The type species is Gallibacter intestinalis.

**(ii) Description of *Gallibacter intestinalis* sp. nov.** *Gallibacter intestinalis* (in.tes.ti.na’lis. N.L. masc. adj. *intestinalis*, pertaining to the intestine). The species has all features of the genus. Cells are approximately 1 μm long and 0.3 μm wide. They grow under strictly anaerobic conditions in GAM modified medium (DSMZ medium 1715) at 37°C. All functional attributes of this species can be found at https://github.com/thh32/Protologger within the data sets section. The major cellular fatty acids were C_15:0 ISO_ (14.9%) and C_15:0 ANTEISO_ (14.7%). Other fatty acids included C_18:1 cis9_ (7.4%), C_16:0 ISO DMA_ (7.4%), C_16:0 DMA_ (6.9%), C_18:2 cis9,12_ (4.4%), C_15:0 ISO DMA_ (3.9%), C_17:0 ANTEISO DMA_ (2.8%), C_16:0_ (2.7%), C_14:0_ (2.5%), and C_16:1 cis9_ (2.2%). The type strain Cla-CZ-54^T^ (= DSM 108706^T^) was isolated from colon content of a free-range layer chicken. Its G+C content of genomic DNA is 40.9%.

**(iii) Description of *Gallalistipes* gen. nov.** *Gallalistipes* (Gall.a.li.sti'pes. L. masc. n. *gallus*, chicken; N.L. masc. n. *Alistipes*, a bacterial genus; N.L. masc. n. *Gallalistipes*, a relative of *Alistipes* from chicken). The isolate is phylogenetically placed into the family *Rikenellaceae* (phylum *Bacteroidetes*) with the closest neighbors being Alistipes onderdonkii, Alistipes finegoldii, and Alistipes timonensis based on 16S rRNA sequence identities of 92.5%, 92.3%, and 92.2%, respectively (Fig. S2B). POCP analysis suggests the genome to belong to the genus *Alistipes* with a matching value of 56% to Alistipes indistinctus. However, the 16S rRNA gene identity to Alistipes indistinctus is, at 91.6%, even lower than to the close relatives aforementioned. POCP to the type species of the genus, Alistipes putredinis, was 48.1%, suggesting the creation of a sister genus. This was also supported by GTDB-Tk being unable to assign the input genome to a sequenced genome at the genus and species levels and by the phylogenomic tree (Fig. S2B). Hence, these data indicate that Alistipes indistinctus may have to be reclassified in the future and that the creation of a novel genus is necessary to accommodate the isolate, for which the name *Gallalistipes* is proposed. The type species is Gallalistipes aquisgranensis.

**(iv) Description of *Gallalistipes aquisgranensis* sp. nov.** *Gallalistipes aquisgranensis* (a.quis.gra.nen’sis. M.L. masc. adj. a*quisgranensis*, pertaining to Aachen (Germany), where the bacterium was isolated). The species has all features of the genus. Cell are small rods that are 1 to 2 μm long and 0.3 μm wide. They grow under strictly anaerobic conditions in GAM modified medium (DSMZ medium 1715) at 37°C. All functional attributes of this species can be found at https://github.com/thh32/Protologger within the data sets section. The major cellular fatty acids were C_15:0 ISO_ (46.2%) and C_18:1 cis9_ (37.9%). Other fatty acids included C_17:0 ISO 3OH_ (4.6%) and C_18:1 t9_ (2.3%). The type strain Cla-CZ-119^T^ (= DSM 108975^T^) was isolated from cecal content of an M11-layer chicken. Its G+C content of genomic DNA is 58%.

**(v) Description of *Gemmiger gallinarum* sp. nov.** *Gemmiger gallinarum* (gal.li.na’rum. L. fem. n. *gallina*, hen; L. gen. fem. pl. n. *gallinarum*, of hens). The closest relative is Gemmiger formicilis, the type species of this genus, with a 16S rRNA gene sequence identity of 96.2%. Even though Gemmiger formicilis is placed into the family *Hyphomicrobiaceae* (phylum *Proteobacteria*) according to its current taxonomic lineage, it clearly falls into the family *Oscillospiraceae* based on phylogenetic trees (Fig. S2C) (104). Other relatives included Fournierella massiliensis and Subdoligranulum variabile within the family *Oscillospiraceae*, with 94.8% and 94.7% sequence identities, respectively, which supports the accommodation of the isolate within this family. The highest ANI values were 81.7% to Subdoligranulum variabile (GCF_000157955.1) and 79.7% to both Gemmiger formicilis and Butyricicoccus pullicaecorum, respectively. GTDB-Tk placed the genome of this isolate into the family *Oscillospiraceae* and assigned it to the genus *Gemmiger* but was unable to provide species-level identification. dDDH values to the closest species Subdoligranulum variabile, Gemmiger formicilis, and Fournierella massiliensis were 25.3%, 22.0%, and 20.2%, respectively. Taken together, a novel species within the genus *Gemmiger* is proposed to accommodate the isolate, supported by a POCP value of 61.2% to Gemmiger formicilis. Cells are rod-shaped, 1 to 2 μm long and 0.4 μm wide, and grow under strictly anaerobic conditions in modified BHI medium (DSMZ medium 215c) at 37°C. All functional attributes of this species can be found at https://github.com/thh32/Protologger within the data sets section. The major cellular fatty acids were C_16:0 DMA_ (37.3%) and C_16:0_ (32.6%). Other fatty acids included C_14:0_ (14.3%) and C_16:0 ALDEHYDE_ (7.7%). The type strain Cla-CZ-245^T^ (= DSM 109015^T^) was isolated from cecal content of an M11-layer chicken. Its G+C content of genomic DNA is 59.2%.

**(vi) Description of *Olsenella gallinarum* sp. nov.** Olsenella gallinarum (gal.li.na’rum. L. fem. n. *gallina*, hen; L. gen. fem. pl. n. *gallinarum*, of hens). According to 16S rRNA gene-based phylogeny, this isolate is placed into the family *Atopobiaceae* (phylum *Actinobacteria*) (Fig. S2D). The closest phylogenetic neighbors are Olsenella umbonata, Olsenella profusa, and Olsenella uli (the type species of this genus) with 96.6%, 96.2%, and 95.8% sequence identities, respectively. The highest ANI value was 80.4% to Olsenella scatoligenes (GCF_001494635.1), which is below the species delineation threshold of 95%. The POCP value to Olsenella uli was 54.9%, indicating that the isolate represents a novel species within the genus *Olsenella*, as confirmed by GTDB-Tk. Cell morphology varies from spherical cells with a diameter of approximately 1 μm to small rods that are approximately 1.6 μm long and 0.4 μm wide and form chains. The species grows under strictly anaerobic conditions in modified BHI medium (DSMZ medium 215c) at 37°C. All functional attributes of this species can be found at https://github.com/thh32/Protologger within the data sets section. The major cellular fatty acids were C_14:0_ (33.8%) and C_14:0 DMA_ (30.3%). Other fatty acids included C_14:0 ALDEHYDE_ (11.6%), C_12:0_ (4.7%), C_16:0 DMA_ (4.3%), C_12:0 DMA_ (2.6%), and C_14:0 ALCOHOL_ (2.5%). The type strain Cla-CZ-62^T^ (= DSM 107455^T^) was isolated from colon content of a free-range layer chicken. Its G+C content of genomic DNA is 68%.

**(vii) Description of *Pseudoflavonifractor gallinarum* sp. nov.** Pseudoflavonifractor gallinarum (gal.li.na’rum. L. fem. n. *gallina*, hen; L. gen. fem. pl. n. *gallinarum*, of hens). The 16S rRNA gene-based identification placed the isolate into the family *Oscillospiraceae*, with highest sequence identity (98.2%) to Pseudoflavonifractor capillosus, the type species of the genus (Fig. S2E). Other relatives include Flavonifractor plautii and Intestinimonas butyriciproducens with 97.7% and 95.3% identities, respectively. The highest ANI value was 82.2% to Pseudoflavonifractor capillosus (GCF_000169255.2). The highest POCP value of 56.9% to Pseudoflavonifractor capillosus suggests that the isolate belongs to the genus *Pseudoflavonifractor*. GTDB-Tk assigned the genome of this isolate to the genus *Flavonifractor* and to the species *Flavonifractor* sp900199495. In summary, the different taxonomic delineation values and the phylogeny are somewhat inconsistent for this isolate. We nonetheless propose the creation of a new species within the genus *Pseudoflavonifractor* to accommodate it, but this group of bacteria (*Flavonifractor* and *Pseudoflavonifractor* spp.) will likely have to be reclassified in the near future once additional strains have been isolated. Cells grow as short rods (0.7 to 1.2 μm in length) with a width of approximately 0.4 μm under strictly anaerobic conditions in WCA medium (DSMZ medium 339a) at 37°C. All functional attributes of this species can be found at https://github.com/thh32/Protologger within the data sets section. The major cellular fatty acids were C_14:0_ (29.7%) and C_16:0 DMA_ (28.7%). Other fatty acids included C_14:0 DMA_ (9.5%), C_12:0_ (8.6%), C_18:0 DMA_ (6.1%), C_16:0 ALDEHYDE_ (4.2%), C_16:0_ (3.6%), and C_14:0 ALDEHYDE_ (2.2%). The type strain Cla-CZ-98^T^ (= DSM 107456^T^) was isolated from cecal content of a free-range layer chicken. Its G+C content of genomic DNA is 59.9%.

**(viii) Description of *Ructibacterium* gen. nov.** *Ructibacterium* (Ruc.ti.bac.te’ri.um. L. masc. n. *ructus* belch, burp; Gr. neut. n. *bakterion* a small rod; N.L. neut. n. *Ructibacterium* a belching, rod-shaped bacterium, referring to the production of gas in liquid culture). The genus is phylogenetically placed into the family *Oscillospiraceae* (phylum *Firmicutes*) based on 16S rRNA gene analysis (Fig. S2F). The closest relatives are Acetivibrio cellulolyticus, the type species of this genus, Acetivibrio thermocellus, and Acetivibrio straminisolvens, with 16S rRNA gene sequence identities of 89.7%, 89.6%, and 89.4%, respectively. POCP did not exceed 50% for any of the close relatives. GTDB-Tk placed the genome of this isolate into the family “*Monoglobaceae”* (not validly published) but was unable to provide genus- or species-level assignment. A novel genus, *Ructibacterium*, is proposed within the currently validly named family *Oscillospiraceae* to accommodate the isolate. The type species is Ructibacterium gallinarum.

**(ix) Description of *Ructibacterium gallinarum* sp. nov.** *Ructibacterium gallinarum* (gal.li.na’rum. L. fem. n. *gallina*, hen; L. gen. fem. pl. n. *gallinarum*, of hens). The species has all features of the genus. Cells are small cocci that are approximately 0.6 μm in diameter. They grow under strictly anaerobic conditions in GAM modified medium (DSMZ medium 1715) at 37°C. All functional attributes of this species can be found at https://github.com/thh32/Protologger within the data sets section. The major cellular fatty acids were C_15:0 ISO_ (22.8%) and C_16:0 ISO_ (19.0%). Other fatty acids included C_15:0 ANTEISO_ (12.6%), C_15:0 ISO DMA_ (9.5%), C_17:0 ANTEISO_ (6.6%), C_16:0 ISO DMA_ (5.4%), C_14:0 ISO_ (3.5%), C_15:0 ANTEISO DMA_ (3.5%), and C_17:0 ISO_ (2.0%). The type strain Cla-CZ-49^T^ (= DSM 107454^T^) was isolated from the cecal content of an M11-layer chicken. Its G+C content of genomic DNA is 43.5%.

**(x) Description of *Sellimonas monacensis* sp. nov.** Sellimonas monacensis (mo.na.cen’sis. M.L. neut. n. *Monacum*, Munich, a German city; M.L. fem. adj. *monacensis*, from/of Munich (Germany), referring to the city where the animal facility of the donor chicken was located). Based on 16S rRNA gene phylogeny, the isolate is placed into the family *Lachnospiraceae* (phylum *Firmicutes*) (Fig. S2G). The closest relatives are Faecalicatena contorta (the type species of the genus *Faecalicatena*), Faecalicatena orotica, and Coprococcus comes, with sequence identities of 94.4%, 93.9%, and 93.9%, respectively. FastANI identified the genome as novel, with the best match of 78.3% to Ruminococcus lactaris (GCF_000155205.1). POCP analysis demonstrates a genus separation from *Faecalicatena* with a value of 44.8% to Faecalicatena contorta. The highest POCP values were identified to Dorea longicatena (56.4%) and to Dorea formicigenerans (51.7%), the type species of the genus *Dorea*, but also to Clostridium scindens (54.7%), Clostridium hylemonae (53.1%), and Sellimonas intestinalis (52.9%). The 16S rRNA gene sequence identity between the isolate and the latter species was 93.7%. GTDB-Tk assigned the isolate to “*Dorea phocaeensis”;* however, this species name is not valid. The highest accordance in dDDH value was for “*Lachnoclostridium phocaeense”* Marseille-P3177 with 84.6% and a G+C difference of 0.3%. Although this bacterium was described in 2017 (105), the name “*Lachnoclostridium phocaeense”* is still not valid. Taken together, and also considering the topology of both the 16S rRNA gene-based and phylogenomic trees (Fig. S2G), we suggest the creation of a novel species within the genus *Sellimonas* to accommodate the isolate, for which the name *Sellimonas monacensis* is proposed. Cells are approximately 1 to 2 μm long and 0.5 μm wide. They grow under strictly anaerobic conditions in GAM modified medium (DSMZ medium 1715) at 37°C. All functional attributes of this species can be found at https://github.com/thh32/Protologger within the data sets section. The main cellular fatty acid was C_14:0_ (17.5%). Other fatty acids included C_18:0 DMA_ (14.0%), C_16:0 DMA_ (12.3%), C_16:0_ (11.6%), C_14:0 DMA_ (9.3%), C_12:0_ (4.9%), C_18:1 cis11 DMA_ (3.9%), C_14:0 ALDEHYDE_ (3.8%), C_18:0 ALDEHYDE_ (2.9%), C_16:0 ALDEHYDE_ (2.5%), C_18:1 cis9 DMA_ (2.4%), and C_18:0_ (2.2%). The type strain Cla-CZ-80^T^ (=DSM 108991^T^) was isolated from cecal content of an M11-layer chicken sampled in Munich. Its G+C content of genomic DNA is 50.3%.

### Data availability.

RNA-seq and 16S rRNA gene amplicon data are accessible at the NCBI via accession numbers PRJNA627064 and PRJNA668258, respectively. The accession numbers for all nearly full-length 16S rRNA gene and draft genome sequences generated in the present studies are provided in Table S1 in the supplemental material.

10.1128/mSystems.01300-20.1 FIG S1 Rarefaction curves of chicken trial 1 (MM versus SPF) and chicken trial 2 (SYN) derived from the 16S rRNA gene amplicon sequencing. Download FIG S1, PDF file, 0.6 MB. Copyright © 2021 Zenner et al. 2021 Zenner et al. https://creativecommons.org/licenses/by/4.0/ This content is distributed under the terms of the Creative Commons Attribution 4.0 International license.

10.1128/mSystems.01300-20.2 FIG S2 16S rRNA gene-based phylogenetic (1) and phylogenomic (2) trees and electron micrographs (3) of novel bacterial taxa described within this study. (A) *Gallibacter intestinalis* gen. nov., sp. nov. (B) *Gallalistipes aquisgranensis* gen. nov., sp. nov. (C) *Gemmiger gallinarum* sp. nov. (D) *Olsenella gallinarum* sp. nov. (E) *Pseudoflavonifractor gallinarum* sp. nov. (F) *Ructibacterium gallinarum* gen. nov., sp. nov. (G) *Sellimonas monacensis* sp. nov. Download FIG S2, PDF file, 0.5 MB. Copyright © 2021 Zenner et al. 2021 Zenner et al. https://creativecommons.org/licenses/by/4.0/ This content is distributed under the terms of the Creative Commons Attribution 4.0 International license.

10.1128/mSystems.01300-20.3 FIG S3 Identification of Cla-CZ-1 (= DSM 109009) as a member of the genus Escherichia. Different identification methods are stated and transmission electron microscopy (TEM) negative stains are displayed to verify the presence of flagella. Download FIG S3, PDF file, 0.2 MB. Copyright © 2021 Zenner et al. 2021 Zenner et al. https://creativecommons.org/licenses/by/4.0/ This content is distributed under the terms of the Creative Commons Attribution 4.0 International license.

10.1128/mSystems.01300-20.4 Table S1 Tabular information about the ChiBAC collection, including genus and species name, original strain designation, DSM number, risk group classification, best 16S rRNA gene sequence identity hits by EZBiocloud, phylum and family level, 16S rRNA gene sequence and length, 16S rRNA accession number, prevalence (%) in the chicken gut (against 1,000 samples), and prevalence of isolates with >1% relative abundance, average and maximum relative abundance, genome accession number (if applicable), predicted antimicrobial resistances and genes (if applicable), geographic location of donor chickens, sampling age, isolation source, and culture conditions, including DSMZ medium, temperature, incubation time, and atmospheric conditions. Isolates used for SYN colonization are indicated in purple, novel taxa are indicated in orange. Download Table S1, XLSX file, 0.04 MB. Copyright © 2021 Zenner et al. 2021 Zenner et al. https://creativecommons.org/licenses/by/4.0/ This content is distributed under the terms of the Creative Commons Attribution 4.0 International license.
