# Supplementary material for: Early-Life Immune System Maturation in Chickens Using a Synthetic Community of Cultured Gut Bacteria
Source: mSystems. 2021 May 18;6(3):e01300-20. doi: 10.1128/mSystems.01300-20 (PMC8269260; doi:10.1128/mSystems.01300-20)

Suppl. Fig. S1: Rarefaction curves derived from the 16S rRNA gene amplicon sequencing data

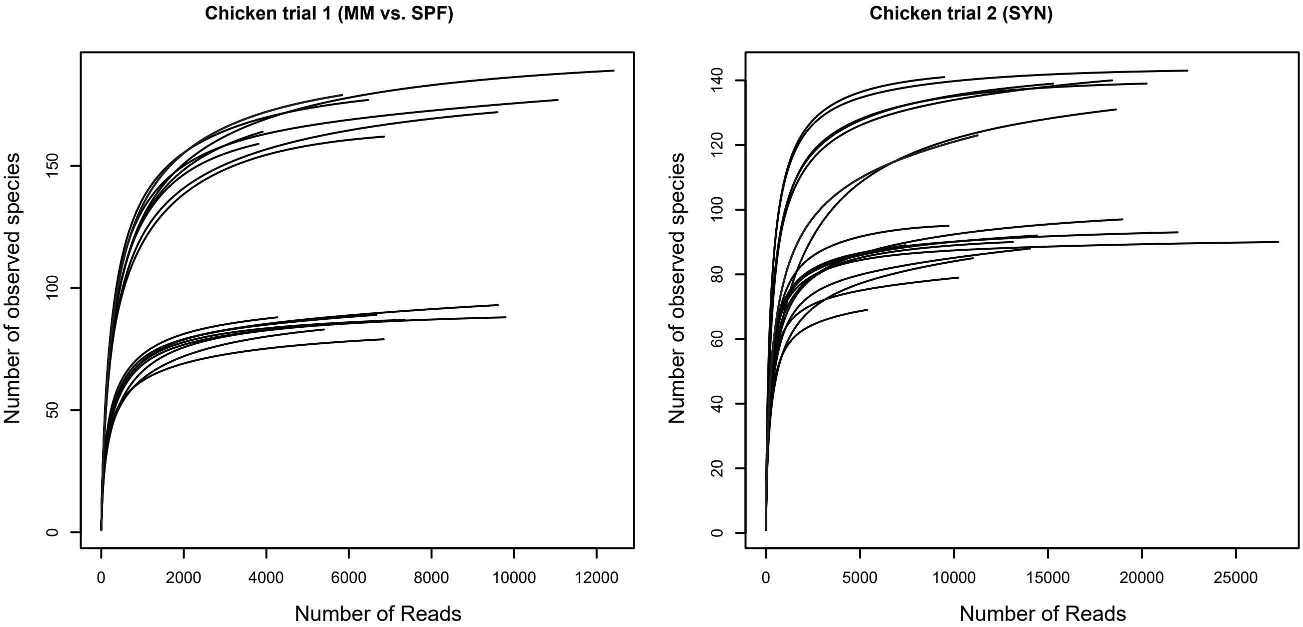

Supplement: FIG S1 [file msystems.01300-20-sf001.pdf]
