# Supplementary material for: Early-Life Immune System Maturation in Chickens Using a Synthetic Community of Cultured Gut Bacteria
Source: mSystems. 2021 May 18;6(3):e01300-20. doi: 10.1128/mSystems.01300-20 (PMC8269260; doi:10.1128/mSystems.01300-20)

Suppl. Fig. S2 A 16S rRNA gene-based phylogenetic (1) and phylogenomic (2) trees; electron micrographs (3) of *Gallibacter intestinalis* gen.nov., sp.nov.

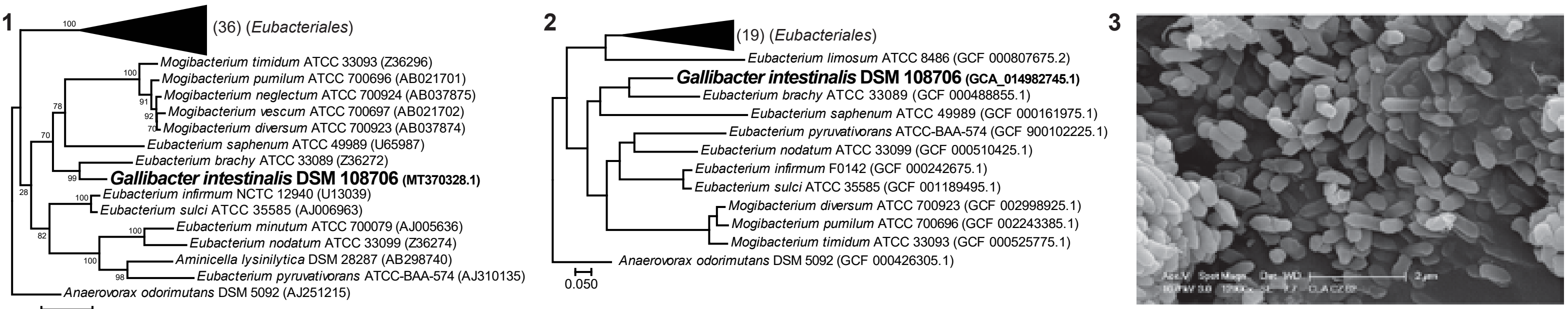

Suppl. Fig. S2 B 16S rRNA gene-based phylogenetic (1) and phylogenomic (2) trees; electron micrographs (3) of *Gallalistipes aquisgranensis* gen.nov., sp.nov.

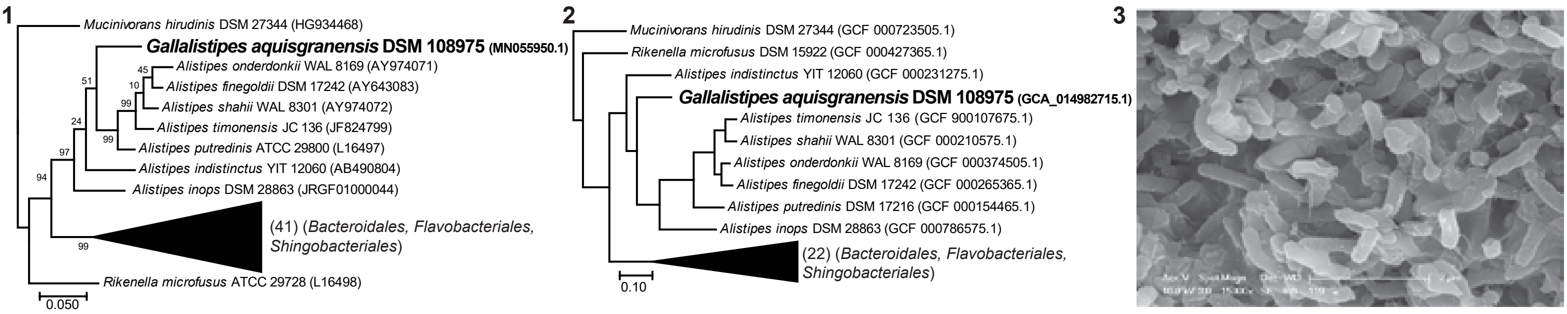

Suppl. Fig. S2 C 16S rRNA gene-based phylogenetic (1) and phylogenomic (2) trees; electron micrographs (3) of *Gemmiger gallinarum* sp.nov.

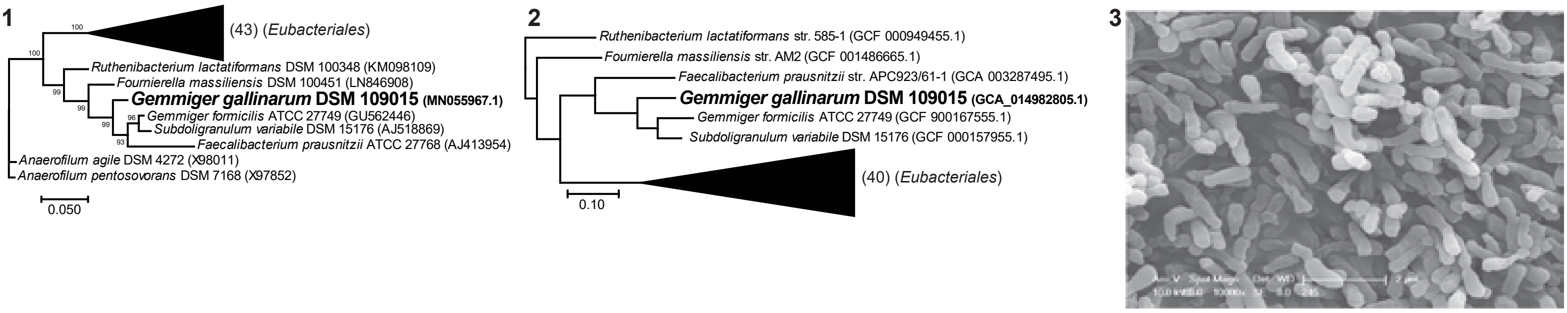

Suppl. Fig. S2 D 16S rRNA gene-based phylogenetic (1) and phylogenomic (2) trees; electron micrographs (3) of *Olsenella gallinarum* sp.nov.

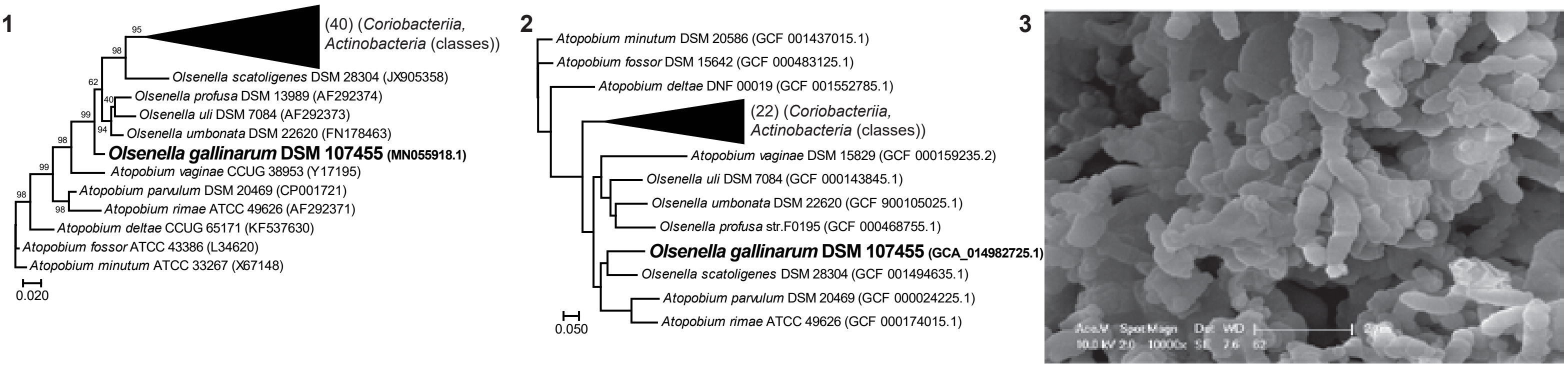

Suppl. Fig. S2 E 16S rRNA gene-based phylogenetic (1) and phylogenomic (2) trees; electron micrographs (3) of *Pseudoflavonifractor gallinarum* sp.nov.

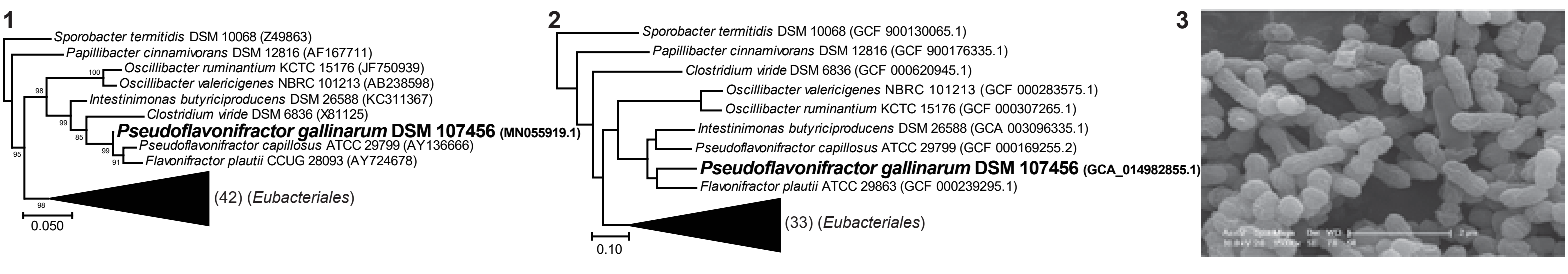

Suppl. Fig. S2 F 16S rRNA gene-based phylogenetic (1) and phylogenomic (2) trees; electron micrographs (3) of *Ructibacterium gallinarum* gen.nov, sp.nov.

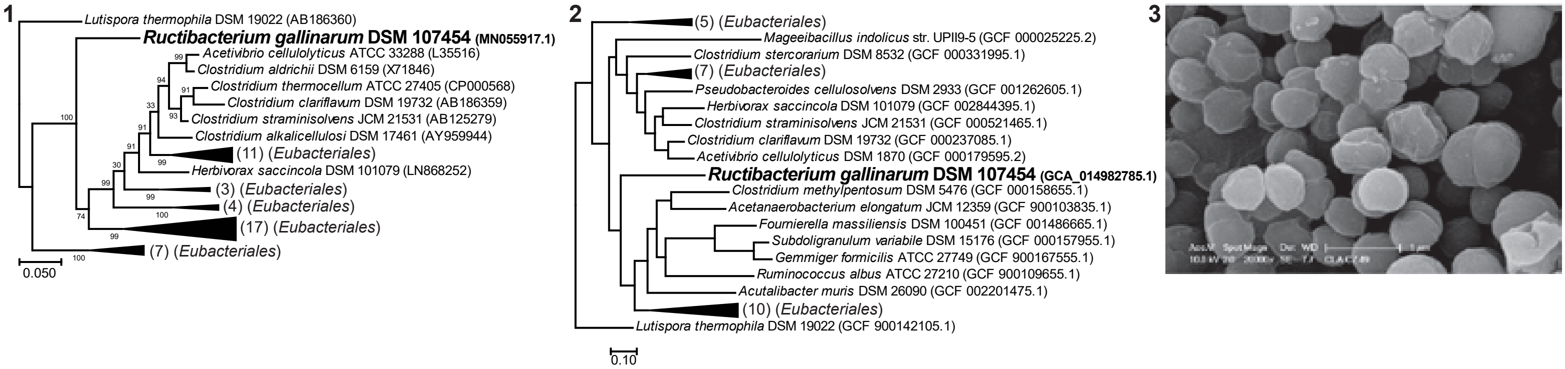

Suppl. Fig. S2 G 16S rRNA gene-based phylogenetic (1) and phylogenomic (2) trees; electron micrographs (3) of *Sellimonas monacensis* sp.nov.

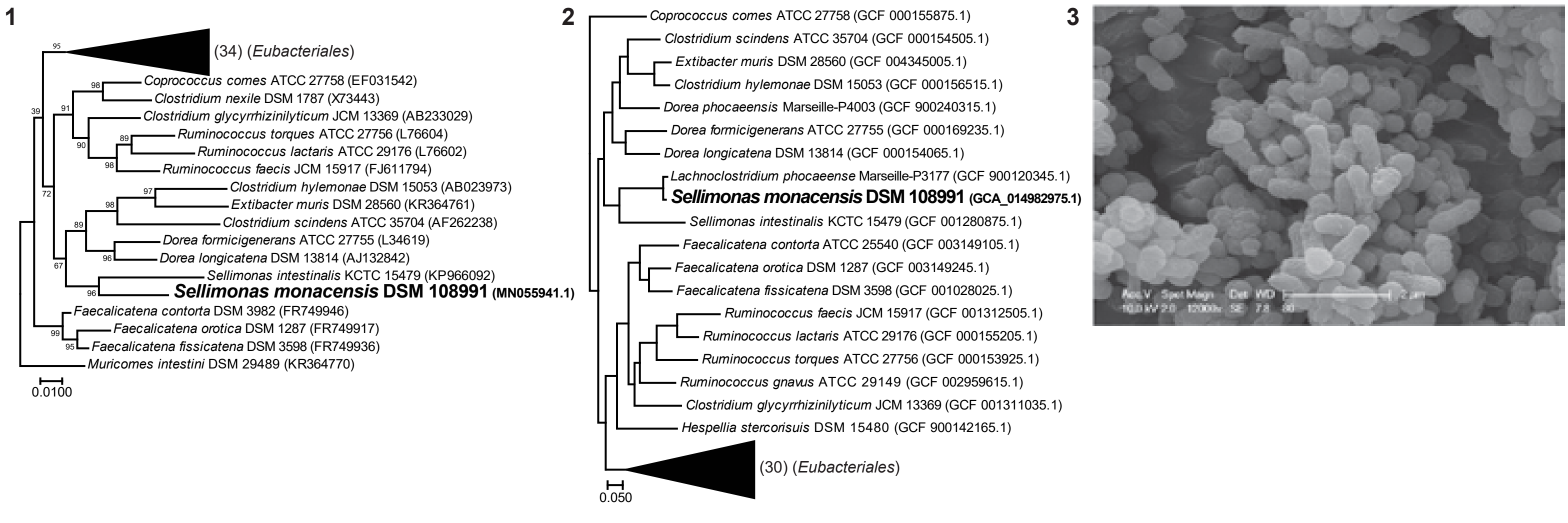

Supplement: FIG S2 [file msystems.01300-20-sf002.pdf]
