# Supplementary material for: Early-Life Immune System Maturation in Chickens Using a Synthetic Community of Cultured Gut Bacteria
Source: mSystems. 2021 May 18;6(3):e01300-20. doi: 10.1128/mSystems.01300-20 (PMC8269260; doi:10.1128/mSystems.01300-20)
Supplement: FIG S3 [file msystems.01300-20-sf003.pdf]

Suppl. Fig. S3: Identification of strain Cla-CZ-1 (=DSM 109009) as a member of the genus *Escherichia*

Identification methods:

MALDI-TOF
Escherichia coli

16S rRNA gene sequence identities
Escherichia marmotae 99.3%
Shigella flexneri 99.2%
Shigella dysenteriae 99.1%
Escherichia coli 99.0%

ANI values
Shigella flexneri 97.8%
Shigella dysenteriae 97.0%
Escherichia coli 96.6%

GTDB-Tk assignment
Escherichia flexneri

dDDH values
Shigella sonnei 88.5%
Shigella boydii 88.4%
Shigella flexneri 85.7%
Shigella dysenteriae 81.8%
Escherichia coli 73.8%
Escherichia marmotae 43.3%

G+C difference
Escherichia coli 0.15%
Shigella flexneri 0.17%
Escherichia marmotae 0.18%

TEM negative stain of strain Cla-CZ-1
Escherichia spp. are usually motile thanks to flagella whilst *Shigella* spp. are not, although their flagella genes may be expressed under rare circumstances
[Girón, Mol Microbiol., 1995 Oct, 18: (1):63-75. doi: 10.1111/j.1365-2958.1995.mmi_18010063.x.]
[Ragupathi et al., New Microbes New Infect, 2018 Jan, 21: 58-62, doi: 10.1016/j.nmni.2017.09.003]

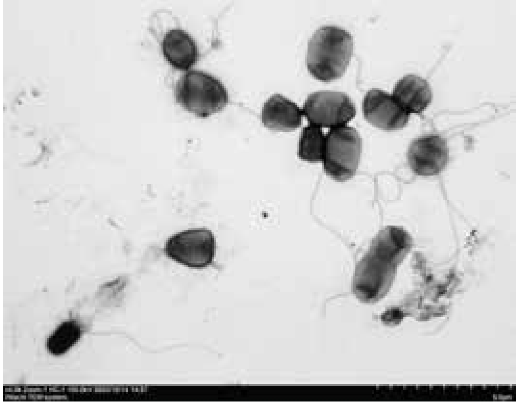
4,000x magnification

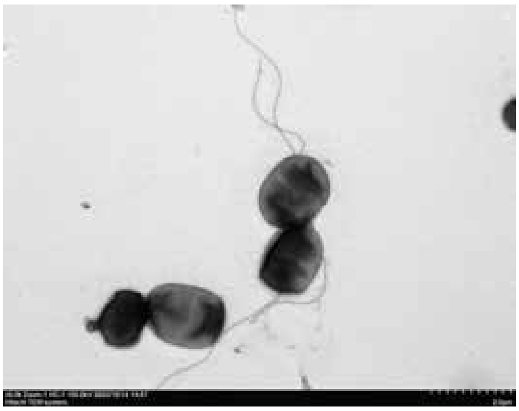
6,000x magnification

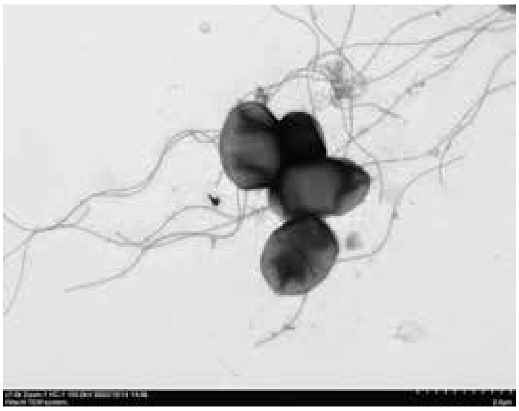
7,000x magnification

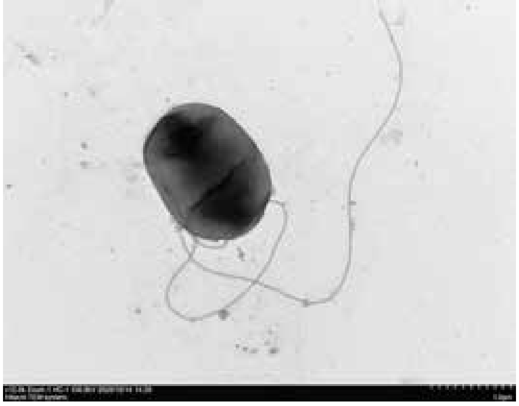
12,000x magnification
